# Pre-Exposure Prophylaxis Integration into Family Planning Services at Title X Clinics in the Southeastern United States: A Geographically-Targeted Mixed Methods Study (Phase 1 ATN 155)

**DOI:** 10.2196/12774

**Published:** 2019-06-11

**Authors:** Jessica M Sales, Cam Escoffery, Sophia A Hussen, Lisa B Haddad, Ashley Phillips, Teresa Filipowicz, Maria Sanchez, Micah McCumber, Betty Rupp, Evan Kwiatkowski, Matthew A Psioda, Anandi N Sheth

**Affiliations:** 1 Department of Behavioral Sciences and Health Education Rollins School of Public Health Emory University Atlanta, GA United States; 2 Department of Gynecology and Obstetrics School of Medicine Emory University Atlanta, GA United States; 3 Collaborative Studies Coordinating Center Department of Biostatistics University of North Carolina at Chapel Hill Chapel Hill, NC United States

**Keywords:** HIV, pre-exposure prophylaxis, implementation science, women's health

## Abstract

**Background:**

Black adolescent and young adult women (AYAW) in the Southern United States are disproportionately affected by HIV. Pre-exposure prophylaxis (PrEP) is an effective, scalable, individual-controlled HIV prevention strategy that is grossly underutilized among women of all ages and requires innovative delivery approaches to optimize its benefit. Anchoring PrEP delivery to health services that AYAW already trust, access routinely, and deem useful for their sexual health may offer an ideal opportunity to reach women at risk for HIV and to enhance their PrEP uptake and adherence. These services include those of family planning (FP) providers in high HIV incidence settings. However, PrEP has not been widely integrated into FP services, including Title X-funded FP clinics that provide safety net sources of care for AYAW. To overcome potential implementation challenges for AYAW, Title X clinics in the Southern United States are uniquely positioned to be focal sites for conceptually informed and thoroughly evaluated PrEP implementation science studies.

**Objective:**

The aim of this study is to assess inner and outer context factors (barriers and facilitators) that may influence the adoption of PrEP prescription and treatment services in Title X clinics serving AYAW in the Southern United States.

**Methods:**

Phase 1 of Planning4PrEP is an explanatory sequential, mixed methods study consisting of a geographically-targeted Web-based survey of Title X clinic administrators and providers in the Southern United States, followed by key informant interviews among a purposively selected subset of responders to more comprehensively assess inner and outer context factors that may influence adoption and implementation of PrEP in Title X FP clinics in the South.

**Results:**

Phase 1 of Planning4PrEP research activities began in October 2017 and are ongoing. To date, survey and key informant interview administration is near completion, with quantitative and qualitative data analysis scheduled to begin soon after data collection completion.

**Conclusions:**

This study seeks to assess inner and outer contextual factors (barriers and facilitators) that may influence the adoption and integration of PrEP prescription and treatment services in Title X clinics serving AYAW in the Southern United States. Data gained from this study will inform a type 1 hybrid effectiveness implementation study, which will evaluate the multilevel factors associated with successful PrEP implementation while evaluating the degree of PrEP uptake, continuation, and adherence among women seen in Title X clinics.

**International Registered Report Identifier (IRRID):**

DERR1-10.2196/12774

## Introduction

Women of childbearing age comprise a majority of adults living with HIV globally. They account for 20% of the 40,000+ new infections in the United States every year, with disproportionate impact on adolescent and young adult women (AYAW) in the South [1]. Black women in the South are disproportionately affected by HIV: 1 in 48 black women is diagnosed with HIV over their lifetime, nearly 20 times the risk for white women [2]. Southern states account for nearly half of new HIV diagnoses despite having only 37% of the population [3]. Effective prevention efforts tailored to the needs of AYAW are therefore needed not only to curb the epidemic among women but also to protect their sexual partners and prevent perinatal infection. Furthermore, scalable approaches that utilize individual-controlled prevention tools are required, as many AYAW are unable to successfully negotiate mutual monogamy or condom use and are unaware of their partner’s HIV status [4-6]. Pre-exposure prophylaxis (PrEP) is an effective [7,8], scalable, and individual-controlled HIV prevention strategy that is underutilized among women of all ages and requires innovative delivery approaches to optimize its benefit [9].

The few available studies among US women report low knowledge and awareness of PrEP [9]. For example, in a US, multi-site study conducted in 2014, less than 10% of women at risk for HIV had heard of PrEP, but once informed, most women found the option to be attractive [10]. Although Centers for Disease Control and Prevention (CDC) estimates that 176,670 US women may benefit from PrEP to prevent sexual HIV acquisition [11], its use among US women remains low [12]. Despite CDC’s clinical guidance for offering PrEP to individuals at substantial risk, data from a national prescription drug database suggest that women, individuals younger than 25 years, and residents of the South have lower levels of PrEP use relative to new HIV diagnoses [13]. Thus, innovative delivery approaches are required to optimize access to PrEP for AYAW, particularly in the Southern United States.

Anchoring PrEP delivery to health services that AYAW already trust, access routinely, and deem useful for their sexual health is of great appeal, as it may offer an ideal opportunity to reach women at risk for HIV and enhance their PrEP uptake and adherence. Family planning (FP) clinics in high HIV incidence settings may be ideal PrEP delivery settings as they are accessed by sexually active women of childbearing age and already provide sexual health services, including HIV testing and prevention counseling. Rather than standard primary care or sexually transmitted infection clinics, most (60%) AYAW utilize FP clinics for sexual health and preventative services [14], and they are viewed with trust among this group [10]. Importantly, shared decision making, a framework promoted in the Quality Family Planning recommendations [15] used by FP providers, is ideal for identifying AYAW at substantial risk of HIV and offering them comprehensive HIV prevention services, including PrEP. Shared decision making is a process in which clinicians and patients work together to make decisions about care (eg, birth control) based on clinical evidence that balances risks and expected outcomes with patient preferences and values**.** However, not all FP providers provide services to large enough numbers of AYAW at high risk of HIV to justify the potential costs associated with preparing for on-site PrEP provision and monitoring. Therefore, efforts to integrate PrEP in FP services should focus on clinics with the highest anticipated impact.

Specifically, Title X-funded FP clinics may be an ideal setting for integrating PrEP into FP services given that they (1) are important safety net sources of care for AYAW, (2) serve clients at risk for HIV infection, and (3) are expected to offer HIV prevention services as part of Quality Family Planning recommendations. The Title X National Family Planning Program provides grants to health department or county hospital–based programs, non-profit stand-alone clinics, and community health clinics such as federally qualifying health centers. Title X supports an extensive network of approximately 4000 nationwide service sites that serve over 4 million clients, 90% of whom were women, and over two-thirds of whom are younger than 30 years [16]. The program is designed to ensure access to contraception, particularly for low-income individuals, but serves as the usual source of medical care for the majority of female clients [17]. Title X clinics serve as safety net providers, particularly in regions without Medicaid expansion [18], which closely overlap with regions that would most benefit from expansion of HIV prevention services [13].

Despite its appeal as an effective, individual-controlled HIV prevention strategy, PrEP has not been widely integrated into FP services in the United States, or specifically, in Title X clinics in the Southern United States. A 2015 national survey of FP providers in the United States, many of whom were Title X clinic providers, found low PrEP knowledge and use; FP providers in the South had lower PrEP knowledge than those in the Northeast or West [19]. Only one-third of respondents could correctly define PrEP and its efficacy, and less than 5% had ever prescribed PrEP. The majority felt uncomfortable prescribing PrEP because of lack of training, revealing an additional challenge to PrEP delivery for women, especially in the South. Although this study showed high provider willingness to prescribe, little is known about provider education and training needs as well as the prioritization, capacity, barriers, and facilitators to integrate PrEP across clinical settings, including Title X funded clinics.

Knowledge gaps exist that prevent optimal implementation of PrEP in real world settings for AYAW in the United States. Limited PrEP implementation science research has been published to date, and most PrEP demonstration projects and implementation studies in the United States have not included cis-gender women [20]. Limited available data suggest that significant implementation challenges exist, particularly for AYAW. Data from a recent (2013-2016) study highlighted missed opportunities for PrEP delivery during care visits that preceded an HIV diagnosis; individuals with missed opportunities for PrEP were more likely to be female, black, and younger than 30 years [21]. A recent PrEP implementation project at a publicly funded community health center in Philadelphia showed that, while more than one-third of potential PrEP clients were women, only 15% of men and 8% of women who expressed interest and were referred ultimately started PrEP [22,23]. Although women were as likely as men to express interest, they were less likely to start, and attrition at each stage of the PrEP engagement process was higher for women [22], suggesting potential unique implementation challenges for women that need to be investigated.

Finally, few models exist describing the organizational processes and strategies associated with successful integration of PrEP delivery in new clinic settings, and none exist specifically for FP clinics [24,25], including those supported by Title X funding. To overcome the aforementioned potential implementation challenges for AYAW, Title X clinics in the Southern United States are uniquely positioned to be focal sites for conceptually informed and thoroughly evaluated PrEP implementation science studies in the United States because (1) FP providers in these clinics may more readily adapt skills used in contraceptive counseling and provision (ie, shared decision making) to PrEP counseling and provision [15]; (2) they are a regular, trusted source of care for AYAW with HIV risk, including black AYAW [10]; (3) they routinely screen and make referrals for intimate partner violence [26] and other known barriers to adherence [27]; and (4) there are virtually no data on PrEP implementation among US women [24]. To address multiple gaps in our understanding of how to optimally provide PrEP and support its use among AYAW in the Southern United States, we devised a multiphase study (Phase 1 study and Phase 2 study). Phase 1 is a mixed methods assessment of Title X clinics across the South to ascertain critical elements of the inner and outer contexts of various Title X clinics relevant for integrating PrEP into FP services. Phase 2 is a hybrid type 1 effectiveness implementation study in 3 Atlanta Title X clinics to evaluate multilevel factors associated with PrEP reach, level of adoption, and implementation (eg, HIV testing and risk assessment screening and PrEP counseling and prescription) within and across clinics, while also thoroughly evaluating the effect on PrEP uptake, continuation, and adherence over a 6-month follow-up period.

The Adolescent Medicine Trials Network for HIV/AIDS Interventions (ATN) is a research program that aims to defeat the rising HIV epidemic among adolescents and young adults in the United States. The overarching goal of the ATN is to increase awareness of HIV status in youth and, for those diagnosed with HIV, increase access to health care. The ATN develops and conducts behavioral, community-based, translational, therapeutic, microbicide, and vaccine trials in youth who are at risk for or living with HIV, with a focus on the inclusion of minors. Our study (ATN 155) is funded as part of the ATN. The combined findings and resulting tools and trainings will be valuable for PrEP integration in Title X-funded or similarly structured FP clinics and could inform future interventions to optimize PrEP delivery for AYAW. In this paper, we describe the research protocol for the Phase 1 study only.

## Methods

### Study Design

Phase 1 of this study utilizes an explanatory sequential, mixed methods design consisting of geographically-targeted surveys and key informant interviews among clinic administrators and providers in Title X FP clinics in the South. The Consolidated Framework for Implementation Research (CFIR) is used to provide a comprehensive set of constructs associated with effective implementation to facilitate evaluation of inner and outer contextual factors (barriers and facilitators) that may influence the adoption of PrEP prescription and treatment services in Title X clinics serving AYAW in the Southern United States.

### Consent and Institutional Review Board Approval

Phase I of this study has been reviewed and approved by the Emory University Institutional Review Board (IRB# 00098606) and University of North Carolina at Chapel Hill Institutional Review Board (IRB#17-2595). Written consent for the Web-based survey will be obtained for all willing participants before survey start. Written consent is asked within the survey. Survey responses are deidentified to protect participants’ privacy. Participants indicate interest in a follow-up qualitative interview during the consent process. Verbal consent is obtained over the telephone before the start of the qualitative interview.

### Participants

Survey administration targets a convenience sample of approximately n=600 clinic providers and administrators (n=400 providers, n=200 administrators) at Title X FP clinics in the Department of Health and Human Services (DHHS) regions III (Washington District of Columbia, Delaware, Maryland, Pennsylvania, Virginia, West Virginia), IV (Alabama, Florida, Georgia, Kentucky, Mississippi, North Carolina, South Carolina, Tennessee), and VI (Arkansas, Louisiana, New Mexico, Oklahoma, Texas). This sample size was selected based on a previous study of PrEP knowledge and attitudes among FP clinicians [19]. From the pool of respondents who express interest in participating in key informant interviews, a subset of approximately n=60 will be purposefully selected for interviews to ensure broad representation of providers and administrators from each of the DHHS regions.

### Recruitment

Online recruitment of participants is supported by the National Clinical Training Center for Family Planning (NCTCFP). Their assistance includes emailing the survey to a Title X clinic listserv, filtering for recipients from DHHS regions III, IV, and VI. Listserv members will receive 1 to 2 email reminders per month. A total of 7 large-scale emails to the Title X clinic listserv will be sent.

Additional recruitment efforts include an electronic link or banner advertisement for the survey posted on the NCTCFP website, engagement with State Title X Grant holders who oversee Title X funding and implementation in clinics within their state, and in-person recruitment at the biannual NCTCFP national meetings for Title X staff and providers.

Participants interested in taking part in key informant interviews indicate their interest during Web-based survey completion. To evenly represent participant types for these interviews, selection of interviewees is based on the following demographics: DHHS region, clinic type, role in the clinic, and current PrEP delivery in the clinic.

### Incentives

Survey participants are offered compensation for their time with a US $30 Amazon gift card, and those who complete the key informant interviews receive an additional US $50 gift card. Participants provide contact information at survey completion to receive the gift cards.

### Data Collection

Data are collected via a Web-based Qualtrics survey. Participants are aware of the survey’s approximate 20-min duration. Key informant interviews are conducted either in person or via telephone based on participant preference. Interviewees are informed the interview will take approximately 45 min to complete. Surveys (Multimedia Appendix 1) and interviews (Multimedia Appendix 2) include questions aimed at identifying and exploring inner and outer factors that may influence the adoption and integration of PrEP into FP services in Title X clinics in the South.

#### Theoretical Frameworks

The CFIR [28] was selected as the framework through which inner and outer contextual factors (barriers and facilitators) that influence adoption of PrEP prescription and treatment services in Title X clinics serving AYAW in the Southern United States will be assessed. The CFIR provides a menu of constructs that have been identified as important for implementation success [28]. The CFIR captures the complex, multilevel nature of implementation and posits that successful implementation of a new innovation (PrEP delivery in FP clinics) will likely require the use of multiple strategies (eg, training, technical assistance, and an internal champion) at multiple levels of the implementation context. The CFIR comprises 39 constructs organized into 5 domains (*intervention characteristics, outer/inner setting, characteristics of individuals, and process*).

#### Consolidated Framework for Implementation Research Constructs Assessed

Implementation-related constructs are developed from the CFIR [28] guided data collection (both quantitative and qualitative). On the basis of a review of the US-focused PrEP implementation literature [29-40], a subset of the 39 CFIR constructs [41] are selected for their likelihood of being a potential barrier (or facilitator) to implementation and/or having sufficient variation across the units of analysis (eg, clinics) [42]. On the basis of the findings from the limited PrEP implementation literature [29-40], we have selected 17 implementation-focused constructs from the CFIR model to assess in the quantitative (from the Qualtrics survey) and qualitative (from key informant interviews) data collection; these pertain to all 5 CFIR domains. Qualtrics survey items are mapped to these 17 CFIR constructs for analysis. The 17 CFIR constructs targeted for quantitative data analysis are described in Table 1.

**Table 1:** Targeted Consolidated Framework for Implementation Research constructs.

Consolidated Framework for Implementation Research construct		Description of construct	PrEP^a^ specific example
**Intervention characteristics [43,44]**			
	Evidence strength and quality	Stakeholders’ perceptions of the quality and validity of evidence supporting the belief that the intervention will have desired outcomes.	To what extent do you think female patients on PrEP have a decreased risk of acquiring HIV?
	Relative advantage	Stakeholders’ perception of the advantage of implementing the intervention versus an alternative solution.	Advantage to onsite PrEP provision verses referral to off-site PrEP for your patients/staff?
	Trialability	The ability to test the intervention on a small scale in the organization, or partial implementation, and to be able to reverse course (undo implementation) if warranted.	Providing PrEP at my clinic seems possible
	Adaptability	The degree to which an intervention can be adapted, tailored, refined, or reinvented to meet local needs	Are screening guidelines for PrEP tailored for women? Adaptable to Quality Family Planning framework?
	Complexity	Perceived difficulty of implementation, reflected by duration, scope, radicalness, disruptiveness, centrality, and intricacy and number of steps required to implement.	I am confident that I or someone in my clinic can provide risk reduction and medication-adherence counseling to patients on PrEP.
	Cost	Costs of the intervention and costs associated with implementing the intervention including investment, supply, and opportunity costs.	Concerns about whether insurers/Medicaid will cover the cost of PrEP and monitoring
**Outer Setting [43,44] *(ie, outer context, factors external to the organization that may influence implementation)***			
	Patient needs and resources	The extent to which patient needs as well as barriers and facilitators to meet those needs are accurately known and prioritized by the organization.	PrEP is compatible with the needs of patients at my clinic.
	Cosmopolitan	The degree to which an organization is networked with other external organizations.	Individuals in my clinic are connected with other community organizations that provide HIV prevention services to patients.
	Peer pressure	Mimetic or competitive pressure to implement an intervention; typically because most or other key peer or competing organizations have or will be implementing intervention.	Other doctors (clinics) in my specialty area will prescribe PrEP to at-risk HIV-negative individuals in the next year.
**Inner setting [43,44] *(ie, inner context, factors internal to the organization that may influence implementation)***			
	Implementation climate [14]	The absorptive capacity for change, shared receptivity of involved individuals to an intervention, and the extent to which use of that intervention will be rewarded, supported, and expected within their organization.	Leadership values evidence-based HIV practices such as PrEP
	Networks and communications	The nature and quality of webs of social networks and the nature and quality of formal and informal communications within an organization.	My clinic works effectively together as a team with community organizations to promote HIV prevention practices in our community.
	Compatibility	The degree of tangible fit between meaning and values attached to the intervention by involved individuals; how those align with individuals’ own norms, values, and perceived risks and needs; and how the intervention fits with existing workflows and systems.	PrEP seems like a good match for patients at my clinic.
	Leadership engagement	Commitment, involvement, and accountability of leaders and managers with the implementation.	My clinic manager would be supportive of PrEP implementation
	Relative priority	Individuals’ shared perception of the importance of the implementation within the organization.	This is a high priority area for Title X clinics in my region.
	Readiness for implementation	Tangible and immediate indicators of organizational commitment to its decision to implement an intervention.	Do you think PrEP education is an essential part of HIV prevention education at family planning visits?
	Available resources	The level of resources dedicated for implementation and on-going operations, including money, training, education, physical space, and time.	We have the necessary support in terms of budget or financial resources
**Characteristics of Individuals [43,44]**			
	Knowledge [19]	Individuals’ beliefs and value placed on the intervention as well as familiarity with facts, truths, and principles related to the intervention.	Before taking this survey, were you aware of Centers for Disease Control and Prevention guidance on PrEP?
	Self-efficacy [15]	Individual belief in their own capabilities to execute courses of action to achieve implementation goals.	I am confident that I can identify individuals at-risk for HIV infection with assistance from an HIV risk screener.
	Attitudes	Individuals’ attitudes toward the intervention.	It is more suitable to provide PrEP in sexually transmitted disease clinics than in family planning clinics.
**Process [43,44]**			
	Executing	Carrying out or accomplishing the implementation according to plan.	Providing HIV test results within 1 week of testing
	Implementation strategies [16]	Most implementation frameworks, including the Exploration, Preparation, Implementation, Sustainment Framework, have 4 components in common: planning (training, tools), engaging (champions, implementation teams), executing, and reflecting and evaluating (monitoring and deciding about continuation/refinements).	The last time you integrated a new method (such as intrauterine devices) into your services, please describe the steps taken to implement that practice at your clinic.

^a^PrEP: pre-exposure prophylaxis.

### Quantitative Data Analysis

The primary outcome from quantitative data analysis for the Phase 1 study is the CFIR *Inner Setting: Readiness for Implementation* construct. The analysis end point is a semi-continuous composite score derived from 19 and 23 Likert-scale survey items for clinic providers and administrators, respectively.

Analyses of the primary outcome will evaluate associations with the following key secondary construct outcomes: (1) *Inner Setting: Implementation Climate*, (2) *Characteristics of Individuals: Knowledge and Beliefs*, (3) *Characteristics of Individuals: Self-Efficacy*, (4) *Inner Setting: Leadership Engagement, and* (5) *Inner Setting: Available Resources*, to explore drivers of implementation readiness. Each of these secondary construct outcomes are semi-continuous composite scores derived from collections of related Qualtrics survey items.

Analyses of primary and secondary construct outcomes will be performed using generalized linear mixed models that account for their being multiple respondents from the same clinic and will adjust for potential confounders including race/ethnicity of the respondent, age, ability to prescribe medication (ie, PrEP), years worked at the clinic, primary role at the clinic, HIV prevalence in the clinic’s catchment area, and census track data linked to the respondents clinic as relevant for the respective analysis.

Full details on the statistical analysis plan (SAP) for quantitative data are provided in the SAP provided in Multimedia Appendix 2 to this paper.

### Qualitative Data Analysis

For coding purposes, “Clinic Type” is considered a case in our qualitative analysis of key informant interviews. We selected this as our “case” as the findings from our study may be especially informative for the provision of Title X support for PrEP scale-up in the clinics that may systematically vary based upon their clinic type (health department/hospital, where multiple health services are available but not always coordinated on-site or during a single visit; community health centers, where multiple health services may be available on-site and same day but specialized expertise may be lacking; and stand-alone FP clinics, where specialized FP services are available on-site but other health services may not be readily available). Coding of the interview will follow a content analysis and deductive approach [45], using the CFIR to guide coding. We will remain open to new themes that may arise inductively from the data as well. Our coding process will follow a consensual research approach, where multiple judges are used throughout the data analysis to ensure multiple perspectives, then consensual validation is achieved through a process of deliberation and consensus among judges, and then an individual “external” to the team (an outside qualitative expert) will review the process to maximize validity of the findings [46]. After the codebook is finalized, the qualitative coding will be conducted in 3 phases: (1) Organize data by CFIR codes and build foundation for case-based analysis, (2) Using Nvivo 11(QSR International Pty Ltd), a pair of analysts will code transcripts and meet to reach consensus then final codes applied for each transcript, and (3) Pairs of analysts will draft a case memo, organized by constructs. The case will be developed iteratively as each transcript is coded, added to, and used to refine the memo. Rigor for qualitative research will be employed by having verbatim transcripts, structured codebook and coding training, double coding, and team consensus on data themes [47,48].

## Results

Phase 1 of Planning4PrEP research activities began in October 2017 and are ongoing. To date, survey and key informant interview administration is near completion, with quantitative and qualitative data analysis scheduled to begin soon after data collection completion.

## Discussion

Although FP clinics may be an ideal setting for PrEP delivery, there is a lack of available data from health care providers and administrators to guide optimal integration of PrEP into various clinical settings, and in particular, for women’s health care settings [24,25]. These data are critical to improve PrEP access and delivery for women. Data gained from this study will facilitate the development of general and context-specific logic models to guide implementation for the adoption of the innovation (PrEP) in a new setting (Title X-funded FP clinics). Furthermore, these data are needed to develop PrEP implementation plans across women’s health care settings and to allow for gathering future data from women on PrEP uptake, adherence, and continuation to develop future interventions to support women’s successful use of PrEP.

## Appendix

Multimedia Appendix 1 Survey items for primary outcome.

Multimedia Appendix 2 Statistical analysis plan for integrating PrEP into family planning services at title X clinics in the Southeastern US-phase 1 (ATN 155).

Multimedia Appendix 3 Lead questions from provider key informant interview guide (for non-PrEP providing clinics)–excludes sub-questions and probes.

## Abbreviations

ATN: Adolescent Medicine Trials Network for HIV/AIDS Interventions
AYAW: adolescent and young adult women
CDC: Centers for Disease Control and Prevention
CFIR: Consolidated Framework for Implementation Research
DHHS: Department of Health and Human Services
FP: family planning
NCTCFP: National Clinical Training Center for Family Planning
PrEP: pre-exposure prophylaxis
SAP: statistical analysis plan
