# Supplementary material for: Pre-Exposure Prophylaxis Integration into Family Planning Services at Title X Clinics in the Southeastern United States: A Geographically-Targeted Mixed Methods Study (Phase 1 ATN 155)
Source: JMIR Res Protoc. 2019 Jun 11;8(6):e12774. doi: 10.2196/12774 (PMC7006615; doi:10.2196/12774)
Supplement: Multimedia Appendix 1 [file resprot_v8i6e12774_app1.docx]

**Appendix 1: Survey Items for Primary Outcome**

| **Inner Setting: Readiness for Implementation among Providers** |
| --- |
| 1. Others in my clinic can screen a patient for symptoms of acute HIV. |
| 1. Others in my clinic can assess a patient’s HIV risk using the CDC PrEP guidelines. |
| 1. Others in my clinic can test a patient for HIV. |
| 1. My clinic has the capacity to provide HIV test results within one week of testing |
| 1. Others in my clinic can assess a patient’s readiness for PrEP. |
| 1. Others in my clinic can assess a patient’s kidney function. |
| 1. My clinic has the capacity to conduct lab work to assess a patients kidney function and provide results within one week of testing |
| 1. Others in my clinic can test a patient for active hepatitis B virus (HBV) infection and interpret results. |
| 1. My clinic has the capacity to provide HBV test results within one week of testing |
| 1. Others in my clinic can ensure a patient is not taking any concomitant medications that may affect their ability to take PrEP. |
| 1. Others in my clinic can counsel a patient on the potential side effects of PrEP. |
| 1. Others in my clinic can counsel a patient on PrEP adherence. |
| 1. Others in my clinic can assess a patient’s pregnancy intentions and conduct preconception or contraceptive counseling. |
| 1. Others in my clinic can prescribe PrEP to a patient. |
| 1. Others in my clinic can help patients navigate insurance payments regarding PrEP treatment. |
| 1. Others in my clinic can refer patients to experts in PrEP and HIV when necessary. |
| 1. My clinic knows where to access resources for PrEP and HIV education |
| 1. If PrEP is prescribed, others in my clinic can conduct 3-month follow up visits for: Medication adherence counseling and side-effect assessment. |
| 1. If PrEP is prescribed, others in my clinic can conduct 3-month follow up visits for: Laboratory testing (HIV, STI, kidney function, and pregnancy testing) |
| 1. If PrEP is prescribed, others in my clinic can conduct 3-month follow up visits for: Pregnancy intentions and preconception or contraceptive counseling |
| 1. My clinic has an onsite pharmacy or affiliated pharmacy that will carry PrEP. |
| 1. There are community-based organizations or other partners in my community that will help facilitate PrEP access for patients at my clinic. |
