# Supplementary material for: Pre-Exposure Prophylaxis Integration into Family Planning Services at Title X Clinics in the Southeastern United States: A Geographically-Targeted Mixed Methods Study (Phase 1 ATN 155)
Source: JMIR Res Protoc. 2019 Jun 11;8(6):e12774. doi: 10.2196/12774 (PMC7006615; doi:10.2196/12774)
Supplement: Multimedia Appendix 2 [file resprot_v8i6e12774_app2.pdf]

Statistical Analysis Plan

STUDY TITLE: Integrating PrEP into Family Planning Services at Title X Clinics in the Southeast: Phase 1 SAP

ATN PROTOCOL NUMBER (PROTOCOL VERSION): 155 (Phase 1, 1.0)

CO-PI: Jessica M. Sales, PhD

CO-PI: Anandi Sheth, MD, MS

FUNDING AGENCY: National Institute of Child Health and Human Development (NICHD)

AUTHOR: Matthew A. Psioda, PhD

STATISTICAL ANALYSIS PLAN VERSION (DATE): 1.0 (August 23, 2018)

SIGNATURE PAGE

Name	Role	Signature	Date
Jessica Sales	Co-Principal Investigator		8/23/2018
Anandi Sheth	Co-Principal Investigator		8/23/2018
Matthew Psioda	ATN CC Co-Investigator		8/23/2018

TABLE OF CONTENTS

1. LIST OF ABBREVIATIONS 1

2. INTRODUCTION 2

3. STUDY METHODS 3

3.1. STUDY DESIGN 3

3.2. STUDY POPULATION 3

3.3. SAMPLE SIZE INFORMATION 3

3.4. RANDOMIZATION 4

3.5. ANALYSIS POPULATIONS 4

3.5.1. All Respondents 4

3.5.2. Respondents from PrEP Providing Clinics 4

3.5.3. Respondents from Non-PrEP Providing Clinics 4

3.5.4. Respondents who are Healthcare Providers 4

3.5.5. Respondents who are Clinic Administrators 4

3.6. IDENTIFICATION OF UNIQUE CLINICS 5

3.7. PRIMARY ENDPOINT 5

3.8. SECONDARY ENDPOINTS 5

3.9. EXPLORATORY ENDPOINTS 5

3.10. ANALYSIS METHODS 6

3.10.1. Analysis of the Primary Endpoint 6

3.10.1.1. Missing Data 7

3.10.2. Analysis of the Secondary Endpoints 8

3.10.3. Analysis of the Exploratory Endpoints 9

4. STATISTICAL SOFTWARE 13

4.1. PSEUDOCODE FOR PRIMARY ENDPOINT ANALYSIS 13

5. LIST OF TABLES & FIGURES 13

6. TABLE AND FIGURE MOCK-UPS 14

7. REFERENCES 26

8. SAP APPENDIX 28

1. LIST OF ABBREVIATIONS

Term	Abbreviation
Adolescent and young adult women	AYAW
Adolescent Medicine Trials Network for HIV/AIDS Interventions	ATN
Consolidated Framework for Implementation Research	CFIR
Family planning	FP
Human immunodeficiency virus	HIV
Pre-exposure prophylaxis	PrEP
United States	US
US Department of Health & Human Services	DHHS

2. INTRODUCTION

Reducing human immunodeficiency virus (HIV) among women, particularly Black adolescent and young adult women (AYAW) in the Southern United States (US), is an urgent public health priority. Women of childbearing age comprise 20% of the >40,000 new HIV infections in the US every year[1]. Southern states account for nearly half of new HIV diagnoses despite having only 37% of the population[2]. One in 48 Black women are diagnosed with HIV over their lifetime, nearly 20 times the risk for White women[3]. Effective prevention efforts tailored to the needs of AYAW are urgently needed, not only to curb the epidemic among women, but also to protect their sexual partners and prevent perinatal infection. Scalable approaches that utilize female-controlled prevention tools are required to reduce HIV risk, since many AYAW are unable to successfully negotiate mutual monogamy or condom use and are unaware of their partner's HIV risk.

Pre-exposure prophylaxis (PrEP) is an effective, scalable, user-controlled HIV prevention strategy that is grossly underutilized among women of all ages and requires innovative delivery approaches to optimize its benefit. The few available studies among US women report low PrEP knowledge and awareness; for example, in a multi-site study conducted in 2014, <10% of women at risk for HIV had heard of PrEP, but once informed, most women found the option to be attractive[4]. To improve PrEP utilization among AYAW, we propose to anchor PrEP delivery to health services that AYAW already trust, access routinely, and deem useful for their sexual health at US Department of Health & Human Services (DHHS) Title X-funded family planning (FP) clinics. However, PrEP has not been widely integrated into FP services, including Title X clinics, especially in the South. Limited available data suggest that significant implementation challenges exist, particularly for AYAW[5].

The aim of Phase 1 of this study is to conduct an explanatory sequential, mixed methods assessment consisting of a geographically-targeted online survey among clinic administrators and providers to assess inner and outer contextual factors that may influence adoption of PrEP in Southern Title X clinics among varying clinic types. Guided by the Consolidated Framework for Implementation Research (CFIR)[6], this study involves a mixed methods assessment of Title X clinics across the South, with a focus on Southern Adolescent Medicine Trials Network for HIV/AIDS Interventions (ATN) site cities, to ascertain vital PrEP-specific attitudes, barriers, facilitators, and clinic- and provider-level capacity for promoting PrEP integration to scale in Title X clinics.

This statistical analysis plan deals with the analysis of quantitative data collected as a part of the geographically-targeted survey.

3. STUDY METHODS

3.1. Study Design

This study employs an explanatory sequential, mixed methods[7] design consisting of a geographically-targeted online survey among DHHS Title X FP clinic administrators and providers in the South, guided by the CFIR[6]. The CFIR provides a menu of constructs that can be used as a practical guide for systematically assessing potential barriers and facilitators in preparation for implementing PrEP, and aiding the development of general or context-specific logic models to guide implementation planning for the adoption of the innovation in a new setting. It is comprised of 39 constructs organized into 5 domains (*Intervention Characteristics, Inner Setting, Outer Setting, Characteristics of Individuals,* and *Process*). Quantitative survey data were collected to evaluate the CFIR constructs described in **Table 8-1**.

Because FP providers and clinic administrators were both eligible to participate in the online survey but have different roles within their clinics, the study team created a survey that utilized question dependencies so that role-specific questions could be asked of providers only and administrators only. Each survey included key constructs (**Table 8-1**) and captured relevant background information on the survey participant and their clinic including demographics, training history, type of clinic (e.g., health department/ stand-alone/ community clinic), clinic name, and clinic address.

3.2. Study Population

This study included providers and administrators who work at Title X FP clinics in DHHS Regions III, IV and VI. These regions include Alabama, Arkansas, Delaware, District of Columbia, Florida, Georgia, Kentucky, Louisiana, Maryland, Mississippi, New Mexico, North Carolina, Oklahoma, Pennsylvania, South Carolina, Tennessee, Texas, Virginia, and West Virginia.

Participant Inclusion Criteria

1. Family Planning (FP) providers, clinic administrators, or Title X Regional Project Officers or State Title X grant holders,
2. From DHHS Title X Regions III, IV, and VI, and
3. Speak English and are 18 years or older.

Participant Exclusion Criteria

1. Individuals who are not healthcare providers, clinic administrators, or Title X Regional Project Officers or State Title X grant holders from DHHS Regions III, IV, and VI.

3.3. Sample Size Information

This is an exploratory study designed to collect information to inform the design of a larger phase II implementation study. As such, and due to the fact that the burden of data collection is minimal once the survey is created, the sample size for the study was targeted to be 600 with a

goal of obtaining as many completed surveys as possible. The target of 600 was viewed as feasible because a similar approach was successfully employed by Seidman et al. in the only national survey of FP providers on PrEP[8]. During the 12 weeks that their survey was posted, 604 providers were surveyed with 99% completion. Building off of Seidman *et al.*'s proven methods, this study aims to recruit 600 participants (400 providers & 200 clinic administrators) with similarly high completion rates.

3.4. Randomization

Randomization was not used in this study.

3.5. Analysis Populations

All surveys completed by respondents from clinics not within DHHS Title X Regions III, IV, and VI will be excluded from all analyses.

3.5.1. All Respondents

This analysis population includes all respondents who initiated and provided, at minimum, their primary role at the clinic where they work. For the analysis of the primary endpoint, each secondary endpoint, and each exploratory endpoint, analyses will be restricted to respondents who responded to at least one survey item from the set used to define the endpoint. Thus, respondents who do not complete any survey items out of the set used to define a given endpoint will be excluded from the analysis of that endpoint.

3.5.2. Respondents from PrEP Providing Clinics

PrEP providing clinics included clinics for which the survey respondent stated that their clinic currently provided PrEP to patients.

3.5.3. Respondents from Non-PrEP Providing Clinics

Non-PrEP providing clinics included all clinics where the survey respondent did not confirm that their clinic currently provided PrEP to patients.

3.5.4. Respondents who are Healthcare Providers

Healthcare or FP provider was defined as anyone who has the potential ability to prescribe, counsel or screen for PrEP, and thus included physicians, advanced practice providers (midwives, nurse practitioners, and physician assistants), nurses, medical assistants, and health educators.

3.5.5. Respondents who are Clinic Administrators

We defined clinic administrator as anyone who serves in an administrative oversight capacity over the Title X activities in a clinic, and thus included clinic coordinators, program managers, or other titled roles denoting decision-making authority in the Title X services in the clinic.

3.6. Identification of Unique Clinics

We identified unique clinic addresses using SAS/GRAPH PROC GEOCODE and the 2017 release of the US Census Bureau TIGER/Line files created for the SAS/GRAPH PROC GEOCODE street-level geocoding method (TIGER2GEOCODE.sas (Version 14), Oct. 2017). We utilized the street-level geocoding method in the GEOCODE procedure to convert full street addresses to latitude and longitude values. If the street address match failed, then ZIP code matching was performed. For records with addresses that did not initially map using PROC GEOCODE, manual searches were conducted to confirm and correct the clinic address and/or zip code. The updated data was rerun through PROC GEOCODE and a manual review of the matches was conducted to ensure that the clinics mapped correctly and consistently. Clinics with identical addresses were assigned unique clinic IDs.

3.7. Primary Endpoint

The primary endpoint is Inner Setting: Readiness for Implementation. This endpoint is derived as a composite score based on the 19 or 23 (provider vs. administrator versions) survey items listed in **Table 8-2**. A description of this construct is provided in **Table 8-1**. Responses to each of the survey items follow a Likert scale (i.e., Very dissatisfied to Very Satisfied) and are scored from 1 to 5. The readiness for implementation endpoint is defined as the average score from the contributing survey items.

3.8. Secondary Endpoints

The secondary endpoints for this study include the following:

1. Inner Setting: Implementation Climate Score 1 - 2
2. Characteristics of Individuals: Knowledge and Beliefs
3. Characteristics of Individuals: Self-Efficacy
4. Characteristics of Individuals: Attitudes Part 1 - 3
5. Inner Setting: Leadership Engagement Score 1 - 2
6. Inner Setting: Available Resources Part 1 - 2

A description of these constructs are provided in **Table 8-1**. These endpoints are derived as a composite score based on the survey items listed in **Table 8-2**. For secondary endpoints (1,3-6), the responses to each of the survey items follow a Likert scale (i.e., Very dissatisfied to Very Satisfied) and are scored from 1 to 5 and averaged to compute the analysis score. For secondary endpoint (2), the endpoint is defined as the number of correct responses to questions on the use of PrEP.

3.9. Exploratory Endpoints

The exploratory endpoints include the following:

1. Intervention Characteristics: Evidence Strength and Quality
2. Intervention Characteristics: Relative Advantage

3. Intervention Characteristics: Trialability
4. Intervention Characteristics: Complexity
5. Intervention Characteristics: Trialability and Complexity
6. Intervention Characteristics: Cost
7. Outer Setting: Patient Needs and Resources
8. Outer Setting: Cosmopolitan
9. Outer Setting: Peer Pressure
10. Inner Setting: Networks and Communications
11. Inner Setting: Compatibility
12. Process: Executing

A description of these constructs are provided in **Table 8-1**. These endpoints are derived as a composite score based on the survey items listed in **Table 8-2**. The responses to each of the survey items follow a Likert scale (i.e., Very dissatisfied to Very Satisfied) and are scored from 1 to 5 and then averaged to compute a score for each endpoint.

For exploratory endpoint (6), we will derive an alternative version which is based on the count of survey items that reflected cost as being a concern based on a dichotomized Likert scale (e.g. Agree/Strongly Agree vs. Strongly Disagree/Disagree/Neutral).

3.10. Analysis Methods

3.10.1. Analysis of the Primary Endpoint

The semi-continuous primary endpoint will be analyzed among responders from non-PrEP providing clinics using a linear mixed model (LMM) that includes a clinic-specific random effect (i.e., random intercept) to account for there being multiple respondents from the same clinic. Estimation of the LMM will be performed using restricted maximum likelihood methods, and degrees of freedom will be estimated using the method of Kenward and Roger[9].

The linear model will include the following key explanatory variables of interest:

1. CFIR construct scores
    - Inner Setting: Implementation Climate Score 1 - 2
    - Characteristics of Individuals: Knowledge and Beliefs
    - Characteristics of Individuals: Self-Efficacy
    - Inner Setting: Leadership Engagement Score 1 - 2
    - Inner Setting: Available Resources Part 1 - 2

In addition to these variables, to the extent that data allows, analyses will adjust for:

1. Clinic-level characteristics
    - Urbanicity of the respondent’s clinic
    - HIV prevalence for the clinic catchment area based on AIDSVu data (see below)

- Presence of staff on site who assist patients with enrolling in Medicaid/insurance programs/Family Planning Waivers
- Presence of pharmacy on site
- Services provided at clinic (Family planning, primary care, other)
- Sociodemographic data for the clinic catchment area based on US census data

2. Respondent-level characteristics
   - Race/ethnicity
   - Age
   - Ability to prescribe medication
   - Years worked at the clinic
   - Primary role at clinic

AIDSVu utilizes data from Centers for Disease Control and Prevention, local health departments, and prescription analytics, and allows users to explore the HIV epidemic at the state-, county-, and ZIP Code-levels.

3.10.1.1. Missing Data

The primary endpoint is derived from 19 to 23 survey items which are not all observed for all respondents from non-PrEP providing clinics. For the primary endpoint analysis, we will use multiple imputation by chained equations[10] to impute missing responses for survey items from partially complete survey responses using full conditional specifications based on the remaining survey items and other clinic- and patient-level data as appropriate. A total of M=100 complete datasets will be imputed in this manner and the primary endpoint will be derived as described above for each imputed dataset. The analysis strategy described above will be implemented for each of the M datasets and the results will be combined using standard techniques[11] to make inference on covariate effects. Missing covariate data will be imputed using a similar procedure so that each of the M imputed dataset has complete outcome and covariate data. Only participants who responded to >= 1 survey item from the set used to define the primary endpoint will be included in the analysis.

3.10.1.1.1. Sensitivity Analyses of the Primary Endpoint

We will use standard diagnostic tools to assess the appropriateness of the normality assumption (e.g., QQ-plots) and, if approximate normality of the residuals is not tenable, sensitivity analyses will be performed to supplement the primary analysis that do not make that assumption. In particular, scores will be categorized into percentiles and analyses will be performed using ordinal logistic mixed models with clinic-specific random effects. With small cluster size, estimation of generalized linear mixed models (GLMMs) is sometimes problematic[12]. If convergence issues arise when the GLMM is estimated using Gaussian quadrature with 4 quadrature points, a reduced model without a random effect may be fit.

We will tabulate characteristics for respondents who partially or fully complete survey items used to define the primary endpoint and for respondents who terminated the survey prior to completing any survey items used to define the primary endpoint to evaluate whether there is apparent selection-bias regarding who completes these survey items. If the data indicate that there are characteristics associated with survey completion, sensitivity analyses will be conducted to explore the robustness of analysis results to selection-bias.

We will evaluate the disagreement of key clinic-level survey items by computing the number of agreements and disagreements reported for unique clinics with two or more respondents. If disagreements are prevalent, sensitivity analyses will be considered to explore robustness of conclusions to errors in responses to clinic-level survey items.

3.10.2. Analysis of the Secondary Endpoints

The following secondary endpoints will be assessed among respondents from non-PrEP providing clinics:

- Inner Setting: Implementation Climate Score 1
- Inner Setting: Leadership Engagement Score 1
- Inner Setting: Available Resources Part 1
- Characteristics of Individuals: Self-Efficacy
- Characteristics of Individuals: Attitudes Part 3

The following secondary endpoints will be assessed among all respondents and separately for non-PrEP providing and PrEP providing clinics:

- Inner Setting: Implementation Climate Score 2
- Characteristics of Individuals: Knowledge and Beliefs
- Characteristics of Individuals: Attitudes Part 1 - 2
- Inner Setting: Leadership Engagement Score 2
- Inner Setting: Available Resources Part 2

The regression models for secondary endpoints will include the following key explanatory variables of interest:

1. Respondent-level characteristics
    - Provider role at clinic
    - Years worked at the clinic
    - Age
2. Clinic-level characteristics
    - Services provided at clinic (family planning, primary care, other)

In addition to these variables, to the extent that data allows, analyses will adjust for the following:

1. Additional clinic-level characteristics

    - Urbanicity of the respondent’s clinic
    - HIV prevalence for the clinic catchment area based on AIDSVu data
    - Presence of staff on site who assist patients with enrolling in Medicaid/insurance programs/Family Planning Waivers
    - Presence of pharmacy on site
    - Services provided at clinic (Family planning, primary care, other)
    - Sociodemographic data for the clinic catchment area based on US census data
2. Additional respondent-level characteristics
    - Ability to prescribe medication

3.10.3. Analysis of the Exploratory Endpoints

The following exploratory endpoints will be assessed among respondents from non-PrEP providing clinics:

- Intervention Characteristics: Evidence Strength and Quality
- Intervention Characteristics: Relative Advantage
- Intervention Characteristics: Trialability

The regression models for these constructs will include the following key explanatory variables of interest:

1. Respondent-level characteristics
    - Provider role at clinic
    - Years worked at the clinic
    - Age

In addition to these variables, to the extent that data allows, analyses will adjust for:

1. Clinic-level characteristics
    - Urbanicity of the respondent’s clinic
    - Sociodemographic data for the clinic catchment area based on US census data

The following exploratory endpoints will be assessed among respondents from non-PrEP providing clinics:

- Intervention Characteristics: Complexity
- Intervention Characteristics: Trialability and Complexity

The regression models for these constructs will include the following key explanatory variables of interest:

1. Respondent-level characteristics
    - Provider role at clinic
    - Years worked at the clinic
    - Age

2. Clinic-level characteristics
    - Presence of staff on site who assist patients with enrolling in Medicaid/insurance programs/Family Planning Waivers
    - Presence of pharmacy on site
    - Services provided at clinic (Family planning, primary care, other)

In addition to these variables, to the extent that data allows, analyses will adjust for:

1. Additional clinic-level characteristics
    - Urbanicity of the respondent's clinic
    - Sociodemographic data for the clinic catchment area based on US census data

The following exploratory endpoint will be assessed among respondents from non-PrEP providing clinics:

- Intervention Characteristics: Cost

The regression models for these constructs will include the following key explanatory variables of interest:

1. Respondent-level characteristics
    - Provider role at clinic
    - Years worked at the clinic
    - Age
2. Clinic-level characteristics
    - Presence of staff on site who assist patients with enrolling in Medicaid/insurance programs/Family Planning Waivers
    - Presence of pharmacy on site
    - Services provided at clinic (Family planning, primary care, other)

In addition to these variables, to the extent that data allows, analyses will adjust for:

1. Additional clinic-level characteristics
    - Urbanicity of the respondent's clinic
    - Sociodemographic data for the clinic catchment area based on US census data
    - Presence of Medicaid expansion in catchment area

The following exploratory endpoint will be assessed among respondents from non-PrEP providing clinics:

- Outer Setting: Patient Needs and Resources

The regression models for these constructs will include the following key explanatory variables of interest:

1. Respondent-level characteristics
    - Provider role at clinic

- Years worked at the clinic
- Age

2. Clinic-level characteristics
    - Presence of staff on site who assist patients with enrolling in Medicaid/insurance programs/Family Planning Waivers
    - Presence of pharmacy on site
    - Services provided at clinic (Family planning, primary care, other)

In addition to these variables, to the extent that data allows, analyses will adjust for:

1. Additional clinic-level characteristics
    - Urbanicity of the respondent’s clinic
    - HIV prevalence for the clinic catchment area based on AIDSVu data
    - Sociodemographic data for the clinic catchment area based on US census data

The following exploratory endpoints will be assessed among respondents from non-PrEP providing clinics:

- Outer Setting: Cosmopolitan and Outer Setting: Peer Pressure

The regression models for these constructs will include the following key explanatory variables of interest:

1. Respondent-level characteristics
    - Provider role at clinic
    - Years worked at the clinic
    - Age
2. Clinic-level characteristics
    - Services provided at clinic (Family planning, primary care, other)

In addition to these variables, to the extent that data allows, analyses will adjust for:

1. Additional clinic-level characteristics
    - Urbanicity of the respondent’s clinic
    - HIV prevalence for the clinic catchment area based on AIDSVu data
    - Sociodemographic data for the clinic catchment area based on US census data

The following exploratory endpoints will be assessed among respondents from non-PrEP providing clinics:

- Inner Setting: Networks and Communications Score 1 - 2
- Inner Setting: Compatibility

The regression models for these constructs will include the following key explanatory variables of interest:

1. Respondent-level characteristics

- Provider role at clinic
- Years worked at the clinic
- Age

2. Clinic-level characteristics
    - Services provided at clinic (Family planning, primary care, other)

In addition to these variables, to the extent that data allows, analyses will adjust for:

1. Additional clinic-level characteristics
    - Urbanicity of the respondent's clinic
    - HIV prevalence for the clinic catchment area based on AIDSVu data
    - Sociodemographic data for the clinic catchment area based on US census data
    - Presence of staff on site who assist patients with enrolling in Medicaid/insurance programs/Family Planning Waivers
    - Presence of pharmacy on site
2. Additional respondent-level characteristics
    - Ability to prescribe medication

The following exploratory endpoint will be assessed among respondents from PrEP providing clinics:

- Process: Executing

The regression model for this construct will include the following key explanatory variables of interest:

1. Respondent-level characteristics
    - Ability to prescribe PrEP
2. Clinic-level characteristics
    - Services provided at clinic (Family planning, primary care, other)
    - Length of time clinic has been prescribing PrEP to patients

In addition to these variables, to the extent that data allows, analyses will adjust for:

1. Additional clinic-level characteristics
    - Urbanicity of the respondent's clinic
    - HIV prevalence for the clinic catchment area based on AIDSVu data
    - Sociodemographic data for the catchment area of clinics based on US census data
    - Presence of staff on site who assist patients with enrolling in Medicaid/insurance programs/Family Planning Waivers
    - Presence of pharmacy on site
2. Additional respondent-level characteristics
    - Ability to prescribe medication

4. STATISTICAL SOFTWARE

All statistical analysis will be conducted in SAS version 9.4 (Cary, NC).

4.1. Pseudocode for primary endpoint analysis

```
PROC GLIMMIX;
 CLASS [class variables] CLINIC;
 MODEL [primary endpoint] = [key explanatory variables] [clinic-level variables] [respondent-level variables]
                                         / DIST=NORMAL LINK=IDENTITY DDFM=KR2;
 RANDOM intercept / SUBJECT = CLINIC;
RUN;
```

5. LIST OF TABLES & FIGURES

In this section include a complete list of tables and figures to be generated for the analysis.

Number	*Title*	*Population*	*Template*
1.0	*Summary of Planning4PrEP Phase 1 Clinical Survey Completion*	*All Respondents*	*T1*
2.0	*Description of Characteristics for Phase 1 Clinical Survey Respondents by Title X Region*	*All Respondents*	*T2*
3.0	*Description of Characteristics for Phase 1 Clinics by Title X Region*	*Unique Clinics*	*T3*
4.X	*Descriptive Analysis of Construct X Components and Summary Scores*<br><br>*Repeat for each primary, secondary, and exploratory endpoint*	*Varies*	*T4*
5.X	*Descriptive Analysis of Construct X Summary Scores by provider type, urban/rural, and DHHS region*	*Varies*	*T5*
6.X	*Construct X Regression Analysis Results*	*Varies*	*T6*
7.0	*Description of Characteristics for Phase 1 Clinic Survey Respondents by Survey Completion Status*	*Varies*	*T7*
8.0	*Disagreement of Key Clinic-Level Variables as Reported by Respondents from the Same Clinic*	*All Respondents from Non-Unique Clinics*	*T8*

6. TABLE AND FIGURE MOCK-UPS

Table T1:

Summary of Planning4PrEP Phase 1 Clinical Survey Completion

Population: All Respondents

Characteristic	Region III - Philadelphia N (%)	Region IV - Atlanta N (%)	Region VI - Dallas N (%)	Overall N (%)
Agreed to participate in the study?[1]	XX (XX.X)	XX (XX.X)	XX (XX.X)	XX (XX.X)
Survey Complete?[2]				
Yes	XX (XX.X)	XX (XX.X)	XX (XX.X)	XX (XX.X)
Yes, but Incomplete	XX (XX.X)	XX (XX.X)	XX (XX.X)	XX (XX.X)
No	XX (XX.X)	XX (XX.X)	XX (XX.X)	XX (XX.X)
Interested in being contacted for follow-up Interview?[2]				XX (XX.X)
Yes	XX (XX.X)	XX (XX.X)	XX (XX.X)	XX (XX.X)
No	XX (XX.X)	XX (XX.X)	XX (XX.X)	XX (XX.X)
Missing	XX (XX.X)	XX (XX.X)	XX (XX.X)	XX (XX.X)

[1] Percentages are based on the number of participants screened.
[2] Percentages are based on the number of participants starting the survey.

Table T2:

Summary of Characteristics of Phase 1 Clinical Survey Respondents by Title X Region

Population: All Respondents

Characteristic	Statistic	Region III - Philadelphia (N=XX)	Region IV - Atlanta (N=XX)	Region VI - Dallas (N=XX)	Overall (N=XX)
Age of Respondent (in years)[1]	N (#Missing)	XX (XX)	XX (XX)	XX (XX)	XX (XX)
	Mean (SD)	XX.X (XX.XX)	XX.X (XX.XX)	XX.X (XX.XX)	XX.X (XX.XX)
	Median	XX.X	XX.X	XX.X	XX.X
	Min, Max	XX.X, XX.X	XX.X, XX.X	XX.X, XX.X	XX.X, XX.X
	n (%)				
20 – 24		XX (XX.X)	XX (XX.X)	XX (XX.X)	XX (XX.X)
25 – 34		XX (XX.X)	XX (XX.X)	XX (XX.X)	XX (XX.X)
35 – 44		XX (XX.X)	XX (XX.X)	XX (XX.X)	XX (XX.X)
45 – 54		XX (XX.X)	XX (XX.X)	XX (XX.X)	XX (XX.X)
55 – 59		XX (XX.X)	XX (XX.X)	XX (XX.X)	XX (XX.X)
60 – 64		XX (XX.X)	XX (XX.X)	XX (XX.X)	XX (XX.X)
65+		XX (XX.X)	XX (XX.X)	XX (XX.X)	XX (XX.X)
Primary role at clinic / clinic PrEP prescribing status	n (%)				
Administrator / PrEP Provider		XX (XX.X)	XX (XX.X)	XX (XX.X)	XX (XX.X)
Administrator / Non-PrEP Provider		XX (XX.X)	XX (XX.X)	XX (XX.X)	XX (XX.X)
Administrator / Unknown PrEP Provider Status		XX (XX.X)	XX (XX.X)	XX (XX.X)	XX (XX.X)
Provider / PrEP Provider		XX (XX.X)	XX (XX.X)	XX (XX.X)	XX (XX.X)
Provider / Non-PrEP Provider		XX (XX.X)	XX (XX.X)	XX (XX.X)	XX (XX.X)
Provider / Unknown PrEP Provider Status		XX (XX.X)	XX (XX.X)	XX (XX.X)	XX (XX.X)
Years worked in primary role[1]	N (#Missing)	XX (XX)	XX (XX)	XX (XX)	XX (XX)
	Mean (SD)	XX.X (XX.XX)	XX.X (XX.XX)	XX.X (XX.XX)	XX.X (XX.XX)
	Median	XX.X	XX.X	XX.X	XX.X
	Min, Max	XX.X, XX.X	XX.X, XX.X	XX.X, XX.X	XX.X, XX.X
Reported clinic classification	n (%)				
Stand-alone family planning clinic		XX (XX.X)	XX (XX.X)	XX (XX.X)	XX (XX.X)
Health Department		XX (XX.X)	XX (XX.X)	XX (XX.X)	XX (XX.X)
Hospital-Based Clinic		XX (XX.X)	XX (XX.X)	XX (XX.X)	XX (XX.X)
Planned Parenthood		XX (XX.X)	XX (XX.X)	XX (XX.X)	XX (XX.X)
Federally Qualified Health Center (FQHC)		XX (XX.X)	XX (XX.X)	XX (XX.X)	XX (XX.X)
Community Clinic		XX (XX.X)	XX (XX.X)	XX (XX.X)	XX (XX.X)
Other		XX (XX.X)	XX (XX.X)	XX (XX.X)	XX (XX.X)
Missing		XX (XX.X)	XX (XX.X)	XX (XX.X)	XX (XX.X)

Characteristic	Statistic	Region III - Philadelphia (N=XX)	Region IV - Atlanta (N=XX)	Region VI - Dallas (N=XX)	Overall (N=XX)
Services provided by clinic[2]	n (%)				
Family Planning		XX (XX.X)	XX (XX.X)	XX (XX.X)	XX (XX.X)
Primary Care		XX (XX.X)	XX (XX.X)	XX (XX.X)	XX (XX.X)
Other		XX (XX.X)	XX (XX.X)	XX (XX.X)	XX (XX.X)
Missing		XX (XX.X)	XX (XX.X)	XX (XX.X)	XX (XX.X)
Clinic staff who assist patients with enrolling in Medicaid/insurance programs/Family Planning Waivers	n (%)				
Yes		XX (XX.X)	XX (XX.X)	XX (XX.X)	XX (XX.X)
No		XX (XX.X)	XX (XX.X)	XX (XX.X)	XX (XX.X)
I don't know		XX (XX.X)	XX (XX.X)	XX (XX.X)	XX (XX.X)
Missing		XX (XX.X)	XX (XX.X)	XX (XX.X)	XX (XX.X)
Respondent's clinic has a pharmacy on site	n (%)				
Yes		XX (XX.X)	XX (XX.X)	XX (XX.X)	XX (XX.X)
No		XX (XX.X)	XX (XX.X)	XX (XX.X)	XX (XX.X)
I don't know		XX (XX.X)	XX (XX.X)	XX (XX.X)	XX (XX.X)
Missing		XX (XX.X)	XX (XX.X)	XX (XX.X)	XX (XX.X)
Respondent has ability to prescribe medication	n (%)				
Yes, without physician supervision		XX (XX.X)	XX (XX.X)	XX (XX.X)	XX (XX.X)
Yes, with physician supervision		XX (XX.X)	XX (XX.X)	XX (XX.X)	XX (XX.X)
No		XX (XX.X)	XX (XX.X)	XX (XX.X)	XX (XX.X)
Missing		XX (XX.X)	XX (XX.X)	XX (XX.X)	XX (XX.X)
Respondent's clinic currently prescribes PrEP to patients	n (%)				
Yes		XX (XX.X)	XX (XX.X)	XX (XX.X)	XX (XX.X)
No		XX (XX.X)	XX (XX.X)	XX (XX.X)	XX (XX.X)
Unknown		XX (XX.X)	XX (XX.X)	XX (XX.X)	XX (XX.X)
Number of months respondent's clinic has prescribed PrEP to patients[1]	N (#Missing)	XX (XX)	XX (XX)	XX (XX)	XX (XX)
	Mean (SD)	XX.X (XX.XX)	XX.X (XX.XX)	XX.X (XX.XX)	XX.X (XX.XX)
	Median	XX.X	XX.X	XX.X	XX.X
	Min, Max	XX.X, XX.X	XX.X, XX.X	XX.X, XX.X	XX.X, XX.X
	n (%)				
0 – <6		XX (XX.X)	XX (XX.X)	XX (XX.X)	XX (XX.X)
7 – <12		XX (XX.X)	XX (XX.X)	XX (XX.X)	XX (XX.X)
12 – <24		XX (XX.X)	XX (XX.X)	XX (XX.X)	XX (XX.X)
24+		XX (XX.X)	XX (XX.X)	XX (XX.X)	XX (XX.X)

Characteristic	Statistic	Region III - Philadelphia (N=XX)	Region IV - Atlanta (N=XX)	Region VI - Dallas (N=XX)	Overall (N=XX)
Who in the respondent's clinic prescribes PrEP to patients[3]	n (%)				
Myself		XX (XX.X)	XX (XX.X)	XX (XX.X)	XX (XX.X)
Other Providers in My Clinic		XX (XX.X)	XX (XX.X)	XX (XX.X)	XX (XX.X)
Both Myself and Other Providers in My Clinic		XX (XX.X)	XX (XX.X)	XX (XX.X)	XX (XX.X)
Missing		XX (XX.X)	XX (XX.X)	XX (XX.X)	XX (XX.X)
Clinic's 2013 NCHS Urban-Rural County Classification[4]	n (%)				
Large Central Metro		XX (XX.X)	XX (XX.X)	XX (XX.X)	XX (XX.X)
Large Fringe Metro		XX (XX.X)	XX (XX.X)	XX (XX.X)	XX (XX.X)
Medium Metro		XX (XX.X)	XX (XX.X)	XX (XX.X)	XX (XX.X)
Small Metro		XX (XX.X)	XX (XX.X)	XX (XX.X)	XX (XX.X)
Micropolitan (Nonmetropolitan)		XX (XX.X)	XX (XX.X)	XX (XX.X)	XX (XX.X)
Noncore (Nonmetropolitan)		XX (XX.X)	XX (XX.X)	XX (XX.X)	XX (XX.X)
Missing Valid Zipcode		XX (XX.X)	XX (XX.X)	XX (XX.X)	XX (XX.X)
Clinic's State	n (%)				
Alabama		XX (XX.X)	XX (XX.X)	XX (XX.X)	XX (XX.X)
Arkansas		XX (XX.X)	XX (XX.X)	XX (XX.X)	XX (XX.X)
Delaware		XX (XX.X)	XX (XX.X)	XX (XX.X)	XX (XX.X)
District of Columbia		XX (XX.X)	XX (XX.X)	XX (XX.X)	XX (XX.X)
Florida		XX (XX.X)	XX (XX.X)	XX (XX.X)	XX (XX.X)
Georgia		XX (XX.X)	XX (XX.X)	XX (XX.X)	XX (XX.X)
Kentucky		XX (XX.X)	XX (XX.X)	XX (XX.X)	XX (XX.X)
Louisiana		XX (XX.X)	XX (XX.X)	XX (XX.X)	XX (XX.X)
Maryland		XX (XX.X)	XX (XX.X)	XX (XX.X)	XX (XX.X)
Mississippi		XX (XX.X)	XX (XX.X)	XX (XX.X)	XX (XX.X)
New Mexico		XX (XX.X)	XX (XX.X)	XX (XX.X)	XX (XX.X)
North Carolina		XX (XX.X)	XX (XX.X)	XX (XX.X)	XX (XX.X)
Oklahoma		XX (XX.X)	XX (XX.X)	XX (XX.X)	XX (XX.X)
Pennsylvania		XX (XX.X)	XX (XX.X)	XX (XX.X)	XX (XX.X)
South Carolina		XX (XX.X)	XX (XX.X)	XX (XX.X)	XX (XX.X)
Tennessee		XX (XX.X)	XX (XX.X)	XX (XX.X)	XX (XX.X)
Texas		XX (XX.X)	XX (XX.X)	XX (XX.X)	XX (XX.X)
Virginia		XX (XX.X)	XX (XX.X)	XX (XX.X)	XX (XX.X)
West Virginia		XX (XX.X)	XX (XX.X)	XX (XX.X)	XX (XX.X)

Footnotes T2:

1 For continuous variables, number missing, mean, standard deviation (SD), median, minimum (min), and maximum (max) values are reported.

2 More than one service may be selected, so these categories are no mutually exclusive. Responses are considered missing if no response was selected for this question.

3 Denominator of the percent is those who responded Yes to "Does your clinic currently prescribe PrEP to patients?".

4 https://www.cdc.gov/nchs/data_access/urban_rural.htm#2013_Urban-Rural_Classification_Scheme_for_Counties.

Table T3:

Summary of Characteristics for Phase 1 Clinics by Title X Region

Population: Unique Clinics

Characteristic	Statistic	Region III - Philadelphia (N=XX)	Region IV - Atlanta (N=XX)	Region VI - Dallas (N=XX)	Overall (N=XX)
HIV Prevalence for the catchment area of clinic (AIDSVu)					
Women of Reproductive Age (in years) (Census Data)					
13-17 years					
18-34 years					
35-44 years					
Race Data for the catchment area of clinic (Census Data)					
American Indian and Alaska Native					
Asian					
Black					
Native Hawaiian and Other Pacific Islander					
White					
Other					
Multiple					
Ethnicity Data for the catchment area of clinic (Census Data)					
Hispanic or Latino					
Not Hispanic or Latino					
Population in Occupied Housing Units by Tenure (Census Data)					
Owner Occupied Housing Units					
Renter Occupied Housing Units					
Sociodemographic Data for the catchment area of clinic based on US census data (Census Data)					

Table T4:

Descriptive Analysis of Construct X Components and Summary Scores

Population: XXXXXXXXX

Variable	Mean	Std. Dev	Minimum	Lower Quartile	Median	Upper Quartile	Maximum	Cronbach's Alpha
Component 1	X.X	X.XXX	X.XX	X.XX	X.XX	X.XX	X.XX	
.<br>.<br>.	.<br>.<br>.	.<br>.<br>.	.<br>.<br>.	.<br>.<br>.	.<br>.<br>.	.<br>.<br>.	.<br>.<br>.	
Component N	X.X	X.XXX	X.XX	X.XX	X.XX	X.XX	X.XX	
Construct Score	X.X	X.XXX	X.XX	X.XX	X.XX	X.XX	X.XX	XX.X

Table T5:

Descriptive Analysis of Construct X Summary Scores by provider type, urban/rural, and DHHS region

Population: XXXXXXX

		Provider Type		Urban/Rural		DHHS Region		
Construct	Statistic	Provider (N=XX)	Admin. (N=XX)	Urban (N=XX)	Rural (N=XX)	II (N=XX)	III (N=XX)	IV (N=XX)=
Construct 1	n (n missing)	XX (XX)	XX (XX)	XX (XX)	XX (XX)	XX (XX)	XX (XX)	XX (XX)
	Mean (SD)	XX.X (XX.XX)	XX.X (XX.XX)	XX.X (XX.XX)	XX.X (XX.XX)	XX.X (XX.XX)	XX.X (XX.XX)	XX.X (XX.XX)
	Median	XX.X (XX.XX)	XX.X (XX.XX)	XX.X (XX.XX)	XX.X (XX.XX)	XX.X (XX.XX)	XX.X (XX.XX)	XX.X (XX.XX)
	Q1 – Q3	XX.X – XX.X	XX.X – XX.X	XX.X – XX.X	XX.X – XX.X	XX.X – XX.X	XX.X – XX.X	XX.X – XX.X
	Min. - Max	XX.X – XX.X	XX.X – XX.X	XX.X – XX.X	XX.X – XX.X	XX.X – XX.X	XX.X – XX.X	XX.X – XX.X
Construct 2	n (n missing)	XX (XX)	XX (XX)	XX (XX)	XX (XX)	XX (XX)	XX (XX)	XX (XX)
	Mean (SD)	XX.X (XX.XX)	XX.X (XX.XX)	XX.X (XX.XX)	XX.X (XX.XX)	XX.X (XX.XX)	XX.X (XX.XX)	XX.X (XX.XX)
	Median	XX.X (XX.XX)	XX.X (XX.XX)	XX.X (XX.XX)	XX.X (XX.XX)	XX.X (XX.XX)	XX.X (XX.XX)	XX.X (XX.XX)
	Q1 – Q3	XX.X – XX.X	XX.X – XX.X	XX.X – XX.X	XX.X – XX.X	XX.X – XX.X	XX.X – XX.X	XX.X – XX.X
	Min. - Max	XX.X – XX.X	XX.X – XX.X	XX.X – XX.X	XX.X – XX.X	XX.X – XX.X	XX.X – XX.X	XX.X – XX.X
.<br>.<br>.	.<br>.<br>.	.<br>.<br>.	.<br>.<br>.	.<br>.<br>.	.<br>.<br>.	.<br>.<br>.	.<br>.<br>.	.<br>.<br>.

Table T6:

Construct X Regression Analysis Results

Population: XXXXXXX

Covariate	Level	Estimate	Standard Error	95% CI
Intercept	---	XX.X	XX.XX	(XX.X, XX.X)
Categorical 1	Ref: Level A			
	Level B	XX.X	XX.XX	(XX.X, XX.X)
	Level C	XX.X	XX.XX	(XX.X, XX.X)
Categorical 2	Ref: Level A			
	Level B	XX.X	XX.XX	(XX.X, XX.X)
Continuous 1	---	XX.X	XX.XX	(XX.X, XX.X)

Table T7:

Summary of Characteristics for Phase 1 Clinic Survey Respondents by Survey Completion Status

Population: All Respondents

Characteristic	Statistic	Fully Completed Primary Endpoint Survey Items (N=XX)	Partially Completed Primary Endpoint Survey Items (N=XX)	No Completed Primary Endpoint Survey Items (N=XX)	Overall (N=XX)
Age of Respondent (in years)[1]	N (#Missing)	XX (XX)	XX (XX)	XX (XX)	XX (XX)
	Mean (SD)	XX.X (XX.XX)	XX.X (XX.XX)	XX.X (XX.XX)	XX.X (XX.XX)
	Median	XX.X	XX.X	XX.X	XX.X
	Min, Max	XX.X, XX.X	XX.X, XX.X	XX.X, XX.X	XX.X, XX.X
Primary role at clinic / clinic PrEP prescribing status	n (%)				
Administrator / PrEP Provider		XX (XX.X)	XX (XX.X)	XX (XX.X)	XX (XX.X)
Administrator / Non-PrEP Provider		XX (XX.X)	XX (XX.X)	XX (XX.X)	XX (XX.X)
Administrator / Unknown PrEP Provider Status		XX (XX.X)	XX (XX.X)	XX (XX.X)	XX (XX.X)
Provider / PrEP Provider		XX (XX.X)	XX (XX.X)	XX (XX.X)	XX (XX.X)
Provider / Non-PrEP Provider		XX (XX.X)	XX (XX.X)	XX (XX.X)	XX (XX.X)
Provider / Unknown PrEP Provider Status		XX (XX.X)	XX (XX.X)	XX (XX.X)	XX (XX.X)
Years worked in primary role[1]	N (#Missing)	XX (XX)	XX (XX)	XX (XX)	XX (XX)
	Mean (SD)	XX.X (XX.XX)	XX.X (XX.XX)	XX.X (XX.XX)	XX.X (XX.XX)
	Median	XX.X	XX.X	XX.X	XX.X
	Min, Max	XX.X, XX.X	XX.X, XX.X	XX.X, XX.X	XX.X, XX.X
Reported clinic classification	n (%)				
Stand-alone family planning clinic		XX (XX.X)	XX (XX.X)	XX (XX.X)	XX (XX.X)
Health Department		XX (XX.X)	XX (XX.X)	XX (XX.X)	XX (XX.X)
Hospital-Based Clinic		XX (XX.X)	XX (XX.X)	XX (XX.X)	XX (XX.X)
Planned Parenthood		XX (XX.X)	XX (XX.X)	XX (XX.X)	XX (XX.X)
Federally Qualified Health Center (FQHC)		XX (XX.X)	XX (XX.X)	XX (XX.X)	XX (XX.X)
Community Clinic		XX (XX.X)	XX (XX.X)	XX (XX.X)	XX (XX.X)
Other		XX (XX.X)	XX (XX.X)	XX (XX.X)	XX (XX.X)
Missing		XX (XX.X)	XX (XX.X)	XX (XX.X)	XX (XX.X)
Services provided by clinic[2]	n (%)				
Family Planning		XX (XX.X)	XX (XX.X)	XX (XX.X)	XX (XX.X)
Primary Care		XX (XX.X)	XX (XX.X)	XX (XX.X)	XX (XX.X)
Other		XX (XX.X)	XX (XX.X)	XX (XX.X)	XX (XX.X)
Missing		XX (XX.X)	XX (XX.X)	XX (XX.X)	XX (XX.X)

Characteristic	Statistic	Fully Completed Primary Endpoint Survey Items (N=XX)	Partially Completed Primary Endpoint Survey Items (N=XX)	No Completed Primary Endpoint Survey Items (N=XX)	Overall (N=XX)
Clinic staff who assist patients with enrolling in Medicaid/insurance programs/Family Planning Waivers	n (%)				
Yes		XX (XX.X)	XX (XX.X)	XX (XX.X)	XX (XX.X)
No		XX (XX.X)	XX (XX.X)	XX (XX.X)	XX (XX.X)
I don't know		XX (XX.X)	XX (XX.X)	XX (XX.X)	XX (XX.X)
Missing		XX (XX.X)	XX (XX.X)	XX (XX.X)	XX (XX.X)
Respondent's clinic has a pharmacy on site	n (%)				
Yes		XX (XX.X)	XX (XX.X)	XX (XX.X)	XX (XX.X)
No		XX (XX.X)	XX (XX.X)	XX (XX.X)	XX (XX.X)
I don't know		XX (XX.X)	XX (XX.X)	XX (XX.X)	XX (XX.X)
Missing		XX (XX.X)	XX (XX.X)	XX (XX.X)	XX (XX.X)
Respondent has ability to prescribe medication	n (%)				
Yes, without physician supervision		XX (XX.X)	XX (XX.X)	XX (XX.X)	XX (XX.X)
Yes, with physician supervision		XX (XX.X)	XX (XX.X)	XX (XX.X)	XX (XX.X)
No		XX (XX.X)	XX (XX.X)	XX (XX.X)	XX (XX.X)
Missing		XX (XX.X)	XX (XX.X)	XX (XX.X)	XX (XX.X)
Respondent's clinic currently prescribes PrEP to patients	n (%)				
Yes		XX (XX.X)	XX (XX.X)	XX (XX.X)	XX (XX.X)
No		XX (XX.X)	XX (XX.X)	XX (XX.X)	XX (XX.X)
Unknown		XX (XX.X)	XX (XX.X)	XX (XX.X)	XX (XX.X)
Number of months the respondent's clinic has been prescribing PrEP to patients[1]	N (#Missing)	XX (XX)	XX (XX)	XX (XX)	XX (XX)
	Mean (SD)	XX.X (XX.XX)	XX.X (XX.XX)	XX.X (XX.XX)	XX.X (XX.XX)
	Median	XX.X	XX.X	XX.X	XX.X
	Min, Max	XX.X, XX.X	XX.X, XX.X	XX.X, XX.X	XX.X, XX.X
	n (%)				
0 – <6		XX (XX.X)	XX (XX.X)	XX (XX.X)	XX (XX.X)
7 – <12		XX (XX.X)	XX (XX.X)	XX (XX.X)	XX (XX.X)
12 – <24		XX (XX.X)	XX (XX.X)	XX (XX.X)	XX (XX.X)
24+		XX (XX.X)	XX (XX.X)	XX (XX.X)	XX (XX.X)
Who in the respondent's clinic prescribes PrEP to patients[3]	n (%)				
Myself		XX (XX.X)	XX (XX.X)	XX (XX.X)	XX (XX.X)
Other Providers in My Clinic		XX (XX.X)	XX (XX.X)	XX (XX.X)	XX (XX.X)
Both Myself and Other Providers in My Clinic		XX (XX.X)	XX (XX.X)	XX (XX.X)	XX (XX.X)
Missing		XX (XX.X)	XX (XX.X)	XX (XX.X)	XX (XX.X)

Characteristic	Statistic	Fully Completed Primary Endpoint Survey Items (N=XX)	Partially Completed Primary Endpoint Survey Items (N=XX)	No Completed Primary Endpoint Survey Items (N=XX)	Overall (N=XX)
Clinic's 2013 NCHS Urban-Rural County Classification[4]	n (%)				
Large Central Metro		XX (XX.X)	XX (XX.X)	XX (XX.X)	XX (XX.X)
Large Fringe Metro		XX (XX.X)	XX (XX.X)	XX (XX.X)	XX (XX.X)
Medium Metro		XX (XX.X)	XX (XX.X)	XX (XX.X)	XX (XX.X)
Small Metro		XX (XX.X)	XX (XX.X)	XX (XX.X)	XX (XX.X)
Micropolitan (Nonmetropolitan)		XX (XX.X)	XX (XX.X)	XX (XX.X)	XX (XX.X)
Noncore (Nonmetropolitan)		XX (XX.X)	XX (XX.X)	XX (XX.X)	XX (XX.X)
Missing Valid Zipcode		XX (XX.X)	XX (XX.X)	XX (XX.X)	XX (XX.X)
Clinic's State	n (%)				
Alabama		XX (XX.X)	XX (XX.X)	XX (XX.X)	XX (XX.X)
Arkansas		XX (XX.X)	XX (XX.X)	XX (XX.X)	XX (XX.X)
Delaware		XX (XX.X)	XX (XX.X)	XX (XX.X)	XX (XX.X)
District of Columbia		XX (XX.X)	XX (XX.X)	XX (XX.X)	XX (XX.X)
Florida		XX (XX.X)	XX (XX.X)	XX (XX.X)	XX (XX.X)
Georgia		XX (XX.X)	XX (XX.X)	XX (XX.X)	XX (XX.X)
Kentucky		XX (XX.X)	XX (XX.X)	XX (XX.X)	XX (XX.X)
Louisiana		XX (XX.X)	XX (XX.X)	XX (XX.X)	XX (XX.X)
Maryland		XX (XX.X)	XX (XX.X)	XX (XX.X)	XX (XX.X)
Mississippi		XX (XX.X)	XX (XX.X)	XX (XX.X)	XX (XX.X)
New Mexico		XX (XX.X)	XX (XX.X)	XX (XX.X)	XX (XX.X)
North Carolina		XX (XX.X)	XX (XX.X)	XX (XX.X)	XX (XX.X)
Oklahoma		XX (XX.X)	XX (XX.X)	XX (XX.X)	XX (XX.X)
Pennsylvania		XX (XX.X)	XX (XX.X)	XX (XX.X)	XX (XX.X)
South Carolina		XX (XX.X)	XX (XX.X)	XX (XX.X)	XX (XX.X)
Tennessee		XX (XX.X)	XX (XX.X)	XX (XX.X)	XX (XX.X)
Texas		XX (XX.X)	XX (XX.X)	XX (XX.X)	XX (XX.X)
Virginia		XX (XX.X)	XX (XX.X)	XX (XX.X)	XX (XX.X)
West Virginia		XX (XX.X)	XX (XX.X)	XX (XX.X)	XX (XX.X)

Footnotes T7:

1 For continuous variables, number missing, mean, standard deviation (SD), median, minimum (min), and maximum (max) values are reported.
2 More than one service may be selected, so these categories are no mutually exclusive. Responses are considered missing if no response was selected for this question.
3 Denominator of the percent is those who responded Yes to "Does your clinic currently prescribe PrEP to patients?".
4 https://www.cdc.gov/nchs/data_access/urban_rural.htm#2013_Urban-Rural_Classification_Scheme_for_Counties.

Table T8:

Disagreement of Key Clinic-Level Variables between Respondents from the Same Clinic

Population: All Respondents Non-Unique Clinics with Two or More Respondents

Characteristic	Number of Unique Clinics with 2+ Respondents	Agreement between All Responders from Same Clinic (N=XX)	Partially Agreement between Responders from Same Clinic (N=XX)	No Agreement between Responders from Same Clinic (N=XX)
Reported clinic classification	XX	XX (XX)	XX (XX)	XX (XX)
Services provided by clinic[2]	XX	XX (XX)	XX (XX)	XX (XX)
Clinic staff who assist patients with enrolling in Medicaid/insurance programs/Family Planning Waivers	XX	XX (XX)	XX (XX)	XX (XX)
Respondent's clinic has a pharmacy on site	XX	XX (XX)	XX (XX)	XX (XX)
Respondent's clinic currently prescribes PrEP to patients	XX	XX (XX)	XX (XX)	XX (XX)
Number of months the respondent's clinic has been prescribing PrEP to patients[1]	XX	XX (XX)	XX (XX)	XX (XX)
Who in the respondent's clinic prescribes PrEP to patients[3]	XX	XX (XX)	XX (XX)	XX (XX)

7. REFERENCES

1. Centers for Disease Control and Prevention. HIV Surveillance Report, 2015; vol. 27. (2016). Available at: https://www.cdc.gov/hiv/library/reports/hiv-surveillance.html. (Accessed: 14th August 2017)
2. Centers for Disease Control and Prevention. CDC Issue Brief: HIV in the Southern United States. (2016). Available at: https://www.cdc.gov/hiv/pdf/policies/cdc-hiv-in-the-south-issue-brief.pdf. (Accessed: 14th August 2017)
3. Hess K, Hu X, Lansky A, Mermin J, Hall HI. Estimating the lifetime risk of a diagnosis of HIV infection in the United States. Conference on Retroviruses and Opportunistic Infections, Boston, MA, February 22-25, 2016; Abstract #52, and CDC Press Release. Available at: https://www.cdc.gov/nchhstp/newsroom/2016/croi-2016.html.
4. Auerbach, J. D., Kinsky, S., Brown, G. & Charles, V. Knowledge, Attitudes, and Likelihood of Pre-Exposure Prophylaxis (PrEP) Use Among US Women at Risk of Acquiring HIV. *AIDS Patient Care STDs* **29,** 102–110 (2015).
5. AIDS Vaccine Advocacy Coalition. Ongoing and Planned PrEP Demonstration and Implementation Studies. *AVAC* (2016). Available at: https://www.avac.org/resource/ongoing-and-planned-prep-demonstration-and-implementation-studies. (Accessed: 4th August 2017)
6. Damschroder, L. J. *et al.* Fostering implementation of health services research findings into practice: a consolidated framework for advancing implementation science. *Implement. Sci. IS* **4,** 50 (2009).

7. Creswell JW, Plano Clark VL. *Designing and conducting mixed methods research*. (Sage Publishers, 2011).
8. Seidman, D., Carlson, K., Weber, S., Witt, J. & Kelly, P. J. United States family planning providers' knowledge of and attitudes towards preexposure prophylaxis for HIV prevention: a national survey. *Contraception* **93,** 463–469 (2016).
9. Kenward, M. G. & Roger, J. H. Small Sample Inference for Fixed Effects from Restricted Maximum Likelihood. *Biometrics* **53,** 983–997 (1997).
10. van Buuren, S. Multiple imputation of discrete and continuous data by fully conditional specification. *Stat. Methods Med. Res.* **16,** 219–242 (2007).
11. Donald B. Rubin. *Multiple Imputation for Nonresponse in Surveys*. (John Wiley & Sons, Inc., 2008).
12. McNeish, D. M. & Harring, J. R. Clustered data with small sample sizes: Comparing the performance of model-based and design-based approaches. *Commun. Stat. - Simul. Comput.* **46,** 855–869 (2017).
13. CFIR Research Team. Consolidated Framework for Implementation Research (CFIR) Technical Assistance Website. Available at: http://www.cfir.org/. (Accessed: 30th December 2016)
14. Ehrhart, M. G., Aarons, G. A. & Farahnak, L. R. Assessing the organizational context for EBP implementation: the development and validity testing of the Implementation Climate Scale (ICS). *Implement. Sci. IS* **9,** 157 (2014).

15. Sachdev, D. D., Stojanovski, K., Liu, A. Y., Buchbinder, S. P. & Macalino, G. E. Intentions to Prescribe Preexposure Prophylaxis Are Associated With Self-efficacy and Normative Beliefs. *Clin. Infect. Dis. Off. Publ. Infect. Dis. Soc. Am.* **58,** 1786–1787 (2014).

16. Powell, B. J. *et al.* A compilation of strategies for implementing clinical innovations in health and mental health. *Med. Care Res. Rev. MCRR* **69,** 123–157 (2012).

8. SAP APPENDIX

Table 8-1 Constructs Assessed in Phase 1.

**CFIR Construct**	**Description of Construct**	**PrEP Specific Example**
Intervention Characteristics[6,13]		
Evidence Strength and Quality	Stakeholders' perceptions of the quality and validity of evidence supporting the belief that the intervention will have desired outcomes	To what extent do you think female patients on PrEP have a decreased risk of becoming HIV-infected?
Relative Advantage	Stakeholders' perception of the advantage of implementing the intervention versus an alternative solution	Advantage to onsite PrEP provision verses referral to off-site PrEP for your patients/staff?
Trialability[13]	The ability to test the intervention on a small scale in the organization, or partial implementation, and to be able to reverse course (undo implementation) if warranted.	Providing PrEP at my clinic seems possible.
Adaptability	The degree to which an intervention can be adapted, tailored, refined, or reinvented to meet local needs	Are screening guidelines for PrEP tailored for women? Adaptable to QFP framework?
Complexity	Perceived difficulty of implementation, reflected by duration, scope, radicalness, disruptiveness, centrality, and intricacy and number of steps required to implement	I am confident that I or someone in my clinic can provide risk reduction and medication-adherence counseling to patients on PrEP.
Trialability and Complexity	Variable with trialability and complexity items combined	Providing PrEP at my clinic seems doable.
Cost	Costs of the intervention and costs associated with implementing the intervention including investment, supply, and opportunity costs	Concerns about whether insurers/medicaid will cover the cost of PrEP and monitoring
Outer Setting[6,13]	*(i.e., outer context, factors external to the organization that may influence implementation)*	
Patient Needs and Resources[13]	The extent to which patient needs, as well as barriers and facilitators to meet those needs, are accurately known and prioritized by the organization	PrEP is compatible with the needs of patients at my clinic.
Cosmopolitan[13]	The degree to which an organization is networked with other external organizations.	Individuals in my clinic are connected with other community organizations that provide HIV prevention services to patients.
Peer pressure	Mimetic or competitive pressure to implement an intervention; typically because most or other key peer or competing organizations have or will be implementing intervention	Other doctors (clinics) in my specialty area will prescribe PrEP to at-risk HIV-negative individuals in the next year.
Inner Setting[6,13]	*(i.e., inner context, factors internal to the organization that may influence implementation)*	
Implementation Climate[14]	The absorptive capacity for change, shared receptivity of involved individuals to an intervention, and the extent to which use of that intervention will be rewarded, supported, and expected within their organization	Leadership values evidence-based HIV practices such as PrEP
Networks and Communications[13]	The nature and quality of webs of social networks and the nature and quality of formal and informal communications within an organization.	My clinic works effectively together as a team with community organizations to promote HIV prevention practices in our community.

CFIR Construct	Description of Construct	PrEP Specific Example
Compatibility[13]	The degree of tangible fit between meaning and values attached to the intervention by involved individuals, how those align with individuals' own norms, values, and perceived risks and needs, and how the intervention fits with existing workflows and systems.	PrEP seems like a good match for patients at my clinic.
Leadership Engagement[13]	Commitment, involvement, and accountability of leaders and managers with the implementation.	My Clinic Manager would be supportive of PrEP implementation
Relative priority	Individuals' shared perception of the importance of the implementation within the organization	This is a high priority area for Title X clinics in my region.
Readiness for implementation	Tangible and immediate indicators of organizational commitment to its decision to implement an intervention	Do you think PrEP education is an essential part of HIV prevention education at family planning (FP) visits?
Available Resources[13]	The level of resources dedicated for implementation and on-going operations, including money, training, education, physical space, and time.	We have the necessary support in terms of budget or financial resources
Characteristics of Individuals[6,13]		
Knowledge s[8]	Individuals' beliefs and value placed on the intervention as well as familiarity with facts, truths, and principles related to the intervention	Before taking this survey, were you aware of CDC guidance on PrEP?
Self-efficacy[15]	Individual belief in their own capabilities to execute courses of action to achieve implementation goals	I am confident that I can identify individuals at-risk for HIV infection with assistance from an HIV risk screener.
Attitudes	Individuals' attitudes toward the intervention.	It is more suitable to provide PrEP in STD clinics than in family planning clinics.
Process[6,13]		
Executing	Carrying out or accomplishing the implementation according to plan	Providing HIV test results within one week of testing
Implementation strategies[16*]	Most implementation frameworks, including EPIS, have four components in common: planning (training, tools), engaging (champions, implementation teams), executing, and reflecting and evaluating (monitoring and deciding about continuation/refinements).	The last time you integrated a new method (like IUDs) into your services, please describe the steps taken to implement that practice at your clinic.

[*] The implementation strategies construct was not assess using the quantitative survey.

Table 8-2 Survey Questions Associated with CFIR Constructs

Intervention Characteristics: Evidence Strength and Quality

1. I am concerned that PrEP is not effective
2. Even though PrEP was shown to be effective in research trials, it wouldn't work in my clinic
3. Offering PrEP to patients at my clinic would be a less effective strategy for HIV prevention than studies have shown.

Intervention Characteristics: Relative Advantage

1. PrEP would be more effective than interventions we are currently promoting (e.g., consistent condom use, abstinence, monogamy & partner testing) to prevent HIV among patients at our clinic.

Intervention Characteristics: Trialability

1. Providing PrEP at my clinic seems doable.
2. Providing PrEP at my clinic seems implementable.
3. Providing PrEP at my clinic seems possible.

Intervention Characteristics: Complexity

1. Providing PrEP at my clinic seems easy to do.

Intervention Characteristics: Trialability and Complexity (Combined)

1. Providing PrEP at my clinic seems doable.
2. Providing PrEP at my clinic seems implementable.
3. Providing PrEP at my clinic seems possible.
4. Providing PrEP at my clinic seems easy to do.

Intervention Characteristics: Cost

1. Providing PrEP at my clinic requires too many human resources.
2. PrEP is too expensive.
3. My clinic would pay for PrEP care (e.g., labs & visit only) if a patient at my clinic cannot pay for PrEP care.
4. My clinic would only be interested in adopting PrEP if funding was provided for PrEP care.
5. Uncertainty around continuation of federal funding (e.g., Title X, Medicaid, Ryan White, etc) would prohibit our clinic from adopting PrEP.

Outer Setting: Patient Needs and Resources

1. PrEP is compatible with the needs of patients at my clinic.
2. My clinic utilizes community resources to meet the HIV prevention needs of patients.
3. My clinic is aware of community resources regarding HIV prevention that are accessible to patients.

Outer Setting: Cosmopolitan

1. Individuals in my clinic are connected with other community organizations that provide HIV prevention services to patients.

Outer Setting: Peer Pressure

1. Family planning clinics in my community that promote HIV prevention practices (e.g., routine HIV testing, condom use, PrEP, etc.) are seen as leaders in sexual and reproductive healthcare.
2. Family planning clinics in my community that promote HIV prevention practices (e.g., routine HIV testing, condom use, PrEP, etc.) are held in high esteem.
3. Other family planning providers in my community will prescribe PrEP to at-risk HIV-negative individuals in the next year.

Inner Setting: Networks and Communications Score 1

1. My clinic works effectively together as a team with community organizations to promote HIV prevention practices in our community.
2. Individuals in my clinic have a sense of personal responsibility for improving patient care and outcomes
3. Individuals in my clinic cooperate to maintain and improve effectiveness of patient care

Inner Setting: Networks and Communications Score 2

1. Individuals in my clinic have a sense of personal responsibility for improving patient care and outcomes
2. Individuals in my clinic cooperate to maintain and improve effectiveness of patient care

Inner Setting: Implementation Climate Score 1

1. Individuals in my clinic will approve of providers prescribing PrEP to at-risk HIV-negative individuals.
2. My clinic hires individuals who have previously used new types of HIV prevention practices, such as 4th generation HIV testing, opt-out HIV testing, PrEP, and post-exposure prophylaxis (PEP).
3. Individuals working at my clinic value new types of HIV prevention practices, such as 4th generation HIV testing, opt-out HIV testing, PrEP, and PEP.

4. Individuals working at my clinic are flexible enough to integrate new types of HIV prevention practices, such as 4th generation HIV testing, opt-out HIV testing, PrEP, and PEP into routine family planning care.
5. Individuals working at my clinic are open to new types of HIV prevention practices, such as 4th generation HIV testing, opt-out HIV testing, PrEP, and PEP.

Inner Setting: Implementation Climate Score 2

1. Individuals in my clinic are willing to innovate and/or experiment to improve clinical procedures
2. Individuals in my clinic are receptive to change in clinical processes

Inner Setting: Implementation Climate Score 3

1. Individuals in my clinic will approve of providers prescribing PrEP to at-risk HIV-negative individuals.
2. My clinic hires individuals who have previously used new types of HIV prevention practices, such as 4th generation HIV testing, opt-out HIV testing, PrEP, and post-exposure prophylaxis (PEP).
3. Individuals working at my clinic value new types of HIV prevention practices, such as 4th generation HIV testing, opt-out HIV testing, PrEP, and PEP.
4. Individuals working at my clinic are flexible enough to integrate new types of HIV prevention practices, such as 4th generation HIV testing, opt-out HIV testing, PrEP, and PEP into routine family planning care.
5. Individuals working at my clinic are open to new types of HIV prevention practices, such as 4th generation HIV testing, opt-out HIV testing, PrEP, and PEP.
6. Individuals in my clinic are willing to innovate and/or experiment to improve clinical procedures
7. Individuals in my clinic are receptive to change in clinical processes

Inner Setting: Compatibility

1. PrEP seems like a good match for patients at my clinic.
2. Patients at my clinic who are at risk for HIV would really benefit from PrEP.
3. PrEP seems suitable for patients at my clinic.

Inner Setting: Readiness for Implementation among Providers

1. Others in my clinic can screen a patient for symptoms of acute HIV.
2. Others in my clinic can assess a patient's HIV risk using the CDC PrEP guidelines.
3. Others in my clinic can test a patient for HIV.
4. My clinic has the capacity to provide HIV test results within one week of testing
5. Others in my clinic can assess a patient's readiness for PrEP.
6. Others in my clinic can assess a patient's kidney function.
7. My clinic has the capacity to conduct lab work to assess a patients kidney function and provide results within one week of testing
8. Others in my clinic can test a patient for active hepatitis B virus (HBV) infection and interpret results.
9. My clinic has the capacity to provide HBV test results within one week of testing
10. Others in my clinic can ensure a patient is not taking any concomitant medications that may affect their ability to take PrEP.
11. Others in my clinic can counsel a patient on the potential side effects of PrEP.
12. Others in my clinic can counsel a patient on PrEP adherence.
13. Others in my clinic can assess a patient's pregnancy intentions and conduct preconception or contraceptive counseling.
14. Others in my clinic can prescribe PrEP to a patient.
15. Others in my clinic can help patients navigate insurance payments regarding PrEP treatment.
16. Others in my clinic can refer patients to experts in PrEP and HIV when necessary.
17. My clinic knows where to access resources for PrEP and HIV education
18. If PrEP is prescribed, others in my clinic can conduct 3-month follow up visits for: Medication adherence counseling and side-effect assessment.
19. If PrEP is prescribed, others in my clinic can conduct 3-month follow up visits for: Laboratory testing (HIV, STI, kidney function, and pregnancy testing)
20. If PrEP is prescribed, others in my clinic can conduct 3-month follow up visits for: Pregnancy intentions and preconception or contraceptive counseling
21. My clinic has an onsite pharmacy or affiliated pharmacy that will carry PrEP.
22. There are community-based organizations or other partners in my community that will help facilitate PrEP access for patients at my clinic.

Inner Setting: Readiness for Implementation among Administrators

1. Individuals in my clinic can screen a patient for symptoms of acute HIV.
2. Individuals in my clinic can assess a patient's HIV risk using the CDC PrEP guidelines.
3. Individuals in my clinic can test a patient for HIV.

4. My clinic has the capacity to provide HIV test results within one week of testing.
5. Individuals in my clinic can assess a patient's readiness for PrEP.
6. Individuals in my clinic can assess a patient's kidney function.
7. My clinic has the capacity to conduct lab work to assess a patient's kidney function and provide results within one week of testing.
8. Individuals in my clinic can test a patient for active hepatitis B virus (HBV) infection and interpret results.
9. My clinic has the capacity to provide HBV test results within one week of testing.
10. Individuals in my clinic can ensure a patient is not taking any concomitant medications that may affect their ability to take PrEP.
11. Individuals in my clinic can counsel a patient on the potential side effects of PrEP.
12. Individuals in my clinic can counsel a patient on PrEP adherence.
13. Individuals in my clinic can assess a patient's pregnancy intentions and conduct preconception or contraceptive counseling.
14. Individuals in my clinic can prescribe PrEP to a patient.
15. Individuals in my clinic can help patients navigate insurance payments regarding PrEP treatment.
16. Individuals in my clinic can refer patients to experts in PrEP and HIV when necessary.
17. My clinic knows where to access resources for PrEP and HIV education (e.g., online)
18. My clinic has an onsite pharmacy or affiliated pharmacy that will carry PrEP.
19. There are community-based organizations or other partners in my community that will help facilitate PrEP access for patients at my clinic.

Inner Setting: Leadership Engagement Score 1

1. My Clinic Manager would be supportive of PrEP implementation
2. Senior leadership/clinical management in my clinic reward clinical innovation and creativity to improve patient care
3. Senior leadership/clinical management in my clinic solicit opinions of clinical staff regarding decisions about patient care
4. Senior leadership/clinical management in my clinic seek ways to improve patient education and increase patient participation in treatment

Inner Setting: Leadership Engagement Score 2

1. Senior leadership/clinical management in my clinic reward clinical innovation and creativity to improve patient care
2. Senior leadership/clinical management in my clinic solicit opinions of clinical staff regarding decisions about patient care
3. Senior leadership/clinical management in my clinic seek ways to improve patient education and increase patient participation in treatment

Inner Setting: Available Resources Part 1

1. My clinic would only be interested in adopting PrEP if Title X or other funding sources required it.

Inner Setting: Available Resources Part 2

2. We have the necessary support in terms of budget or financial resources
3. We have the necessary support in terms of training
4. We have the necessary support in terms of facilities
5. We have the necessary support in terms of staffing

Characteristics of Individuals: Knowledge (Note: No Alpha, Sum Scores)

1. Pre-exposure prophylaxis (PrEP) is an FDA-approved method for HIV prevention that involves
2. In clinical trials of sexually active adults, among patients who took PrEP as prescribed, the efficacy of PrEP in preventing HIV was
3. Which medication has been FDA-approved for PrEP use?
4. Your patient had a high-risk sexual exposure 3 weeks ago. They want to start PrEP. What is/are the best test(s) to determine their HIV status? (Choose all correct responses).
5. How often should patients on PrEP be followed for medication side effects and lab toxicities after initial assessment?

Characteristics of Individuals: Self-Efficacy

1. I can screen a patient for symptoms of acute HIV.
2. I can assess a patient's HIV risk using the CDC PrEP guidelines.
3. I can test a patient for HIV.
4. I can assess a patient's readiness for PrEP.
5. I can assess a patient's kidney function.

6. I can test a patient for active hepatitis B virus (HBV) infection and interpret results.
7. I can ensure a patient is not taking any concomitant medications that may affect their ability to take PrEP.
8. I can counsel a patient on the potential side effects of PrEP.
9. I can counsel a patient on PrEP adherence.
10. I can assess a patient’s pregnancy intentions and conduct preconception or contraceptive counseling.
11. I can prescribe PrEP to a patient.
12. I can help patients navigate insurance payments regarding PrEP treatment.
13. I can refer patients to experts in PrEP and HIV when necessary.
14. If PrEP is prescribed, I can conduct 3-month follow up visits for: Medication adherence counseling and side-effect assessment.
15. If PrEP is prescribed, I can conduct 3-month follow up visits for: Laboratory testing (HIV, STI, kidney function, and pregnancy testing).
16. If PrEP is prescribed, I can conduct 3-month follow up visits for: Pregnancy intentions and preconception or contraceptive counseling.

Characteristics of Individuals: Attitudes Part 1

1. Do you think HIV prevention education is an essential part of family planning visits?
2. Do you think PrEP education is an essential part of HIV prevention education during family planning visits?

Characteristics of Individuals: Attitudes Part 2

1. It is more suitable to provide PrEP in STD clinics than in family planning clinics.
2. It is more suitable to provide PrEP in clinics that specialize in HIV care than in family planning clinics.
3. The use of PrEP will increase HIV drug resistance.
4. I am concerned that PrEP is not effective.
5. The use of PrEP will result in less federal funding for HIV treatment.
6. Non-biomedical (behavioral) HIV prevention interventions should be attempted before prescribing PrEP.
7. The use of PrEP will cause patients to engage in riskier behaviors.
8. For an HIV-negative patient in a relationship with an HIV-positive partner, treating the HIV-positive partner with antiretroviral therapy (ART) should be attempted instead of prescribing PrEP.
9. For an HIV-negative patient in a relationship with an HIV-positive partner, treating the HIV-positive partner with ART should be attempted before prescribing PrEP.
10. I am concerned about the potential side effects of PrEP.

Characteristics of Individuals: Attitudes Part 3

1. PrEP would have a visible and substantial impact on the health status of patients at my clinic.

Process: Executing

Asked for each of the following: “How difficult was each PrEP prescription activity when you/your clinic FIRST began to prescribe PrEP?

1. Screening a patient for symptoms of acute HIV.
2. Assessing a patient’s HIV risk using the CDC PrEP guidelines.
3. Testing a patient for HIV.
4. Providing HIV test results within one week of testing.
5. Assessing a patient’s readiness for PrEP.
6. Assessing a patient’s kidney function.
7. Providing results of kidney function assessment within one week of testing.
8. Testing a patient for active hepatitis B virus (HBV) infection and interpreting results.
9. Providing HBV test results within one week of testing.
10. Ensuring a patient is not taking any concomitant medications that may affect their ability to take PrEP.
11. Counseling a patient on the potential side effects of PrEP.
12. Counseling a patient on PrEP adherence.
13. Assessing a patient’s pregnancy intentions and conducting preconception or contraceptive counseling.
14. Prescribing PrEP to a patient.
15. Helping patients navigate insurance payments regarding PrEP treatment.
16. Referring patients to experts in PrEP and HIV when necessary.
17. Knowing where to access resources for PrEP and HIV education (e.g., online)
18. Conducting 3-month follow up visits for medication adherence counseling and side-effect assessment.
19. Conducting 3-month follow up visits for laboratory testing (HIV, STI, kidney function, and pregnancy testing).
20. Conducting 3-month follow up visits for pregnancy intentions and preconception or contraceptive counseling.
21. Accessing a pharmacy or affiliated pharmacy that will carry PrEP.

22. Collaborating with community-based organizations or other partners in my community that will help facilitate PrEP access for patients at my clinic.

Process: Facilitators/Barriers

Asked for each of the following: “Has this activity gotten easier, more difficult, or remained the same over time?”

1. Screening a patient for symptoms of acute HIV.
2. Assessing a patient’s HIV risk using the CDC PrEP guidelines.
3. Testing a patient for HIV.
4. Providing HIV test results within one week of testing.
5. Assessing a patient’s readiness for PrEP.
6. Assessing a patient’s kidney function.
7. Providing results of kidney function assessment within one week of testing.
8. Testing a patient for active hepatitis B virus (HBV) infection and interpreting results.
9. Providing HBV test results within one week of testing.
10. Ensuring a patient is not taking any concomitant medications that may affect their ability to take PrEP.
11. Counseling a patient on the potential side effects of PrEP.
12. Counseling a patient on PrEP adherence.
13. Assessing a patient’s pregnancy intentions and conducting preconception or contraceptive counseling.
14. Prescribing PrEP to a patient.
15. Helping patients navigate insurance payments regarding PrEP treatment.
16. Referring patients to experts in PrEP and HIV when necessary.
17. Knowing where to access resources for PrEP and HIV education (e.g., online)
18. Conducting 3-month follow up visits for medication adherence counseling and side-effect assessment.
19. Conducting 3-month follow up visits for laboratory testing (HIV, STI, kidney function, and pregnancy testing).
20. Conducting 3-month follow up visits for pregnancy intentions and preconception or contraceptive counseling.
21. Accessing a pharmacy or affiliated pharmacy that will carry PrEP.
22. Collaborating with community-based organizations or other partners in my community that will help facilitate PrEP access for patients at my clinic.
