# Supplementary material for: Pre-Exposure Prophylaxis Integration into Family Planning Services at Title X Clinics in the Southeastern United States: A Geographically-Targeted Mixed Methods Study (Phase 1 ATN 155)
Source: JMIR Res Protoc. 2019 Jun 11;8(6):e12774. doi: 10.2196/12774 (PMC7006615; doi:10.2196/12774)
Supplement: Multimedia Appendix 3 [file resprot_v8i6e12774_app3.docx]

## Appendix 3: Lead Questions from Provider Key Informant Interview Guide (For Non-PrEP providing clinics) – excludes sub-questions and probes.

## HIV Priority

1. Compared to the other services offered at your clinic, how much of a priority are your HIV prevention services?
2. How much of a problem do you think HIV transmission is for your patients?

## PrEP Priority

1. How much of a priority should providing PrEP related care for HIV prevention be for your clinic?
2. To what extent might the implementation of PrEP take a backseat to other high-priority initiatives going on now?
3. Do you see advantages to providing PrEP services for patients at your clinic? [IF YES] Please describe them.

## Capacity & Implementation

1. How would you describe the overall current ability of your clinic for providing PrEP?
2. Do you have a sense of how many patients at your clinic may be eligible for PrEP?
3. Can you describe how your clinic would go about implementing Step 1 of PrEP services: assessments for HIV risk for patients?
4. Referring to the second step on the sheet, can you describe how your clinic could implement assessing candidacy for PrEP?
5. Referring to the third step on the sheet, can you describe how your clinic would prescribe PrEP?
6. Referring to the fourth step on the sheet, PrEP follow up includes visits every 3 months. Can you describe how your clinic would engage in PrEP monitoring every 3 months?
7. In your clinic, how would you work as a team to implement PrEP services? (Probe: is it like other services you offer?)
8. How much of a concern is time in providing these services on top of the existing schedule?
9. Of the steps we’ve discussed—assessing HIV risk, assessing PrEP eligibility, prescribing PrEP, and PrEP follow up—which ones do you think would be most feasible for your clinic to implement?
   1. Would your clinic need to refer patients to a different location for certain steps?
10. If your clinic was to start providing PrEP services, how long do you think it would take to get it going?

## Resources

1. Considering what we have discussed in the steps above, where do you think your clinic is related to resources for providing PrEP services overall?
2. There are a variety of support roles that may be needed for PrEP implementation, these include insurance related support and navigation, education and counseling, as well as adherence support. Do you have individuals who could fill these roles currently working in the clinic?

## New Practices: Adoption & Decision Making

1. Can you describe what the process at your clinic might be for making a decision to adopt a new practice?
2. How does clinic management/leadership communicate these decisions to adopt new practices with staff/providers?
3. What are key considerations for the decision-making process to adopt new practices at your clinic?

## Champions

1. Are there certain individuals at your clinic who are likely to champion (go above and beyond what might be expected) to support the clinic’s use of EB practices like PrEP?

## Trainings

1. Reflecting on your previous experiences with training in your clinic, what should be considered in terms of training for PrEP implementation overall for your clinic?

## External Factors

1. What external factors, beyond the clinic and its management, might influence your clinic to adopt PrEP services?
2. What external factors, beyond the clinic and its management, might get in the way of adopting PrEP services in your clinic?

## Closing

1. Considering what we discussed today, where do you hope to be in terms of addressing HIV prevention for your patients in the next year?
2. What role should Title X clinics in your state be taking to address HIV, if any?
